# Fish species lifespan prediction from promoter cytosine‐phosphate‐guanine density

**DOI:** 10.1111/1755-0998.13774

**Published:** 2023-03-19

**Authors:** Alyssa M. Budd, Benjamin Mayne, Oliver Berry, Simon Jarman

**Affiliations:** ^1^ School of Biological Sciences The University of Western Australia Perth Western Australia Australia; ^2^ Environomics Future Science Platform, Indian Ocean Marine Research Centre Commonwealth Scientific and Industrial Research Organisation (CSIRO) Crawley Western Australia Australia; ^3^ Curtin University Bentley Perth Western Australia Australia

**Keywords:** CpG, elastic net, fish, longevity, prediction, *t*_max_

## Abstract

Lifespan is a key attribute of a species' life cycle and varies extensively among major lineages of animals. In fish, lifespan varies by several orders of magnitude, with reported values ranging from less than 1 year to approximately 400 years. Lifespan information is particularly useful for species management, as it can be used to estimate invasion potential, extinction risk and sustainable harvest rates. Despite its utility, lifespan is unknown for most fish species. This is due to the difficulties associated with accurately identifying the oldest individual(s) of a given species, and/or deriving lifespan estimates that are representative for an entire species. Recently it has been shown that CpG density in gene promoter regions can be used to predict lifespan in mammals and other vertebrates, with variable accuracy across taxa. To improve accuracy of lifespan prediction in a non‐mammalian vertebrate group, here we develop a fish‐specific genomic lifespan predictor. Our new model includes more than eight times the number of fish species included in the previous vertebrate model (*n* = 442) and uses fish‐specific gene promoters as reference sequences. The model predicts fish lifespan from genomic CpG density alone (measured as CpG observed/expected ratio), explaining 64% of the variance between known and predicted lifespans. The predictions are highly robust to variation in genome quality and are applicable to all classes of fish; a taxonomically diverse and speciose group. The results demonstrate the value of promoter CpG density as a universal predictor of fish lifespan that can applied where empirical data are unavailable, or impracticable to obtain.

## 1 INTRODUCTION

Lifespan is the estimated maximum age that individuals of a given species are expected to attain under favourable environmental conditions. Derivations of a species' lifespan are varied, including the maximum recorded age of any single individual (de Magalhães & Costa, 2009), the age to which a proportion of the population survives (e.g., 5%; Mayne et al., 2020), or, in fish, the age at which 95% of the maximum or asymptotic length is reached (Taylor, 1958). Lifespan derived in any way is a fundamental life history parameter, allowing for approximation of mortality and rate of population growth (Dureuil & Froese, 2021; Then et al., 2015). Lifespan can also provide an upper limit for an animal's reproductive life phase, except in the small number of species that undergo reproductive senescence. The age at which sexual maturity is attained and either age at death or age of reproductive senescence vary more extensively than maximum lifespan, and rates of reproduction and mortality even more so (Healy et al., 2019). Lifespan, in contrast, is a relatively stable trait within a given species and can therefore be used to obtain generalisable information about that species (Austad, 2015; Berkel & Cacan, 2021).

Lifespan's utility in modelling life history makes it valuable for species management. For example, it can be used to quantify sustainable harvest levels for wild populations, such as in fisheries (King & McFarlane, 2003), but also assessments of invasion potential (Tabak et al., 2018), and extinction risk (Bird et al., 2020). Despite its simplicity as a population parameter, and great value for a range of animal population and species management applications, lifespan is often not considered because there are no reliable estimates available. Reported vertebrate lifespans range from 8 weeks in the coral reef pygmy goby (*Eviota sigillata*) (Depczynski & Bellwood, 2005) to approximately 400 years in the Greenland shark (*Somniosus microcephalus*) (Nielsen et al., 2016). Identification of the oldest individuals of a given species is often difficult or unfeasible because age information is sparse or absent. Long‐lived species present a range of practical difficulties for determining lifespan, as in the absence of indirect estimation methods, research programmes rarely last as long as the oldest individuals (Mayne et al., 2020). Thus, despite its central importance to species management and conservation, lifespan is unknown for most animals (de Magalhães & Costa, 2009).

The aging process is thought to be an unintended consequence of cell programming, involving molecular changes that leave traceable genomic signatures (Horvath & Raj, 2018). Consistent changes in a well‐studied epigenetic modification, DNA methylation, can be used to predict individual age in a growing number of species (e.g., Bors et al., 2021; de Paoli‐Iseppi et al., 2019; Horvath & Raj, 2018; Larison et al., 2021; Mayne et al., 2021). This is because, over the lifespan of an individual, global patterns of DNA methylation change, whereby highly methylated regions become demethylated and sparsely methylated regions become methylated (Jung & Pfeifer, 2015). Along with other important epigenetic changes, these changes in DNA methylation result in a loss of cellular functioning that is thought to contribute to processes of aging (Yang et al., 2019). The term DNA methylation is generally used to refer to methylation that occurs at cytosine‐phosphate‐guanine (CpG) sites, or “CG” sequences in the genome, where its occurrence and function has been most extensively studied (Jones, 2012). CpG sites are located throughout the genome but are concentrated around transcription start sites and in promoter regions of genes, where their density and DNA methylation levels are most often associated with changes in gene activity (Sharif et al., 2010). The elevated frequency of CpG sites (i.e., CpG density) in gene promoters has been hypothesised to act as a buffer against age‐related DNA methylation changes and therefore correlate with species maximum lifespan (McLain & Faulk, 2018).

The association between promoter CpG density and lifespan was first revealed in mammals and its predictive value was subsequently demonstrated among all vertebrates (Mayne et al., 2019; McLain & Faulk, 2018). McLain and Faulk (2018) revealed significant correlations between promoter CpG density and mammalian lifespan for 1000 gene promoter regions; 5% of the total examined. Mayne et al. (2019) developed a model that used the CpG densities of 42 gene promoters to predict lifespan in vertebrates, accounting for 76% of the variation between known and predicted lifespans. The vertebrate model highlighted unique relationships between CpG density and lifespan in all major vertebrate groups, including fish, birds, mammals and reptiles. However, the prediction accuracy was lower in non‐mammalian vertebrates, which was attributed to low sample size (*n* ≤ 63) and high sequence divergence (Mayne et al., 2019). The use of human gene promoters as reference sequences in previous lifespan analyses has resulted in fewer sequence matches and lower prediction accuracy in distant relatives (Mayne et al., 2019; McLain & Faulk, 2018). Previous analyses have also obtained lifespan information from the Animal Aging and Longevity Database (AnAge) alone (de Magalhães & Costa, 2009). Although AnAge is a highly comprehensive and well curated database, incorporation of lifespan data from additional sources (e.g., alternative online databases or manual literature search) is likely to increase sample size and improve statistical power.

Fish (aquatic vertebrates with fins and gills) are a paraphyletic group including class Actinopteri (ray‐finned fishes), Chondrichthyes (cartilaginous fishes), Sarcopterygii (fleshy‐finned fishes), Cephalaspidomorphi (e.g., lampreys) and Myxini (e.g., hagfishes). At present, approximately 7000 fish species are subject to wild harvest, each typically requiring species‐specific life history information to enable adequate fisheries management (Froese & Pauly, 2010). An estimated 35% of global fish stocks are now overfished and another 57% are fished at the maximum sustainable yield (FAO, 2022). A lack of data for most fished species is a substantial impediment to the development of sustainable fisheries (Costello et al., 2012). Lifespan data is of particularly high value for management of fish populations, as it can be used to approximate natural mortality rates (Hoenig, 2017), fisheries maximum sustainable yield (Gulland, 1970) and model population growth (Cortés, 2016).

Here, we report the development of a fish‐specific genomic lifespan predictor. The model was constructed using 1804 reported lifespan values for 442 fish species with whole genome sequences available. These genome sequences were used to measure CpG density (measured as CpG observed/expected ratio [see Gardiner‐Garden & Frommer, 1987]) in promoter regions identified using homology to experimentally defined zebrafish (*Danio rerio*) promoter sequences. The model predicts lifespan for any given fish species from the genome sequence of a single individual, demonstrating the high value of promoter CpG density alone to predict lifespan in fish.

## 2 MATERIALS AND METHODS

### 2.1 Known lifespan data collection

A comprehensive dataset of fish lifespan values (including those reported as longevity or maximum age [*t*_max_]) was built by combining information from existing databases, publicly available fisheries data and by conducting a manual literature search (Table S1). To ensure the appropriateness of the complete data set for lifespan prediction, the model was tested using different subsets of the data, and the resulting accuracy compared. The mean of all recorded values for a given species was used as an estimate of known lifespan (referred to as “known lifespan” hereafter) as there was high variability in reported lifespan values. The mean lifespan value was selected as it is more likely to be representative of the lifespan of all individuals of a given species than the measured value of the single oldest individual reported (Dureuil & Froese, 2021). The model was also tested using the median, for completeness.

### 2.2 Genomic data and promoter sequence generation

All available fish genomes were downloaded from the National Centre for Biotechnology Information (NCBI), filtering for classes Actinopteri, Cladista, Chondrichthyes, Cephalaspidomorphi, Hyperoartia, Myxini and Sarcopterygii (see Table S2 for accession numbers). If multiple genome assemblies were available for a species, NCBI's “representative” and “reference” genome classes were used to select the most appropriate assembly for downstream analyses. For species with more than five genome assemblies derived from different individuals available, all assemblies were downloaded and used to assess within‐species variability in lifespan predictions. Genome completeness was assessed using Benchmarking Universal Single‐Copy Orthologs (busco; version 5.2.2), specifying the Actinopterygii lineage dataset (actinopterygii_odb10) and Augustus gene predictor.

Promoter sequences that have been experimentally validated for transcriptional activity in zebrafish were downloaded from the Eukaryotic Promoter Database (EPD) using the EPDnew selection tool (Périer et al., 2000). At present, zebrafish are the only fish species for which EPD promoter sequences are available. For each gene, the region ±100 nucleotides surrounding the transcription start site (TSS) of the most representative gene promoter was extracted. This region was selected as it most probably encompasses the core promoter, a region immediately surrounding the TSS that functions in controlling the activity of RNA polymerase II, and therefore gene transcription (Lenhard et al., 2012). The model was also tested using the default EPD setting (−400 to +100) as well as an extended region covering a peak in CpG density around the TSS in fish (−500 to +1500 bp) (Mayne et al., 2019). As described previously (Mayne et al., 2019; McLain & Faulk, 2018), the EPD promoter sequences were used to query each genome via Basic Local Alignment Search Tool (blast+; version 2.12.0) using a minimum sequence identity of 70%. The single top hit for each promoter in each species was used to calculate CpG observed/expected ratio.

### 2.3 Calculation of CpG observed/expected ratio

The observed/expected ratio of CpGs (CpG O/E) was used as a measure of under‐ or over‐representation of the density of CpG dinucleotides in fish genomes and promoter regions. This measure was developed by (Gardiner‐Garden & Frommer, 1987) to identify CpG islands. CpG O/E is calculated by first obtaining the CpG density (i.e., the total number of CpG dinucleotides [CpG] divided by the sequence length [N]) and dividing it by the expected CpG density, or the C density (i.e., total number of cytosines [C] divided by N) multiplied by the G density (i.e., total number of guanines [G] divided by N) as follows:

$$\mathrm{CpG}\;\text{Observed}/\text{Expected}=\frac{\mathrm{CpG}\;\text{density}}{C\;\text{density}*G\;\text{density}}$$

Is equal to:

$$\mathrm{CpG}\;O/E=\frac{\frac{\mathrm{CpG}}{N}}{\frac{C}{N}\times \frac{G}{N}}$$

Which can be simplified to:

$$\mathrm{CpG}\;O/E=\frac{\mathrm{CpG}}{C\times G}\times N$$

Using this equation, values for CpG O/E were calculated for each promoter sequence and genome in each species. If no matching promoter sequence was obtained during the BLAST search, CpG O/E was given as 0 in the lifespan prediction model.

### 2.4 Lifespan prediction modelling

To predict fish lifespan from CpG O/E, an elastic net regression model was developed using 10‐fold nested cross‐validation in r version 4.1.2 (R Core Team, 2013). First, lifespan values from all fish species with genomic information available were natural log transformed to enable the data to fit a linear model. Based on the percentiles of the transformed values, the data was then split 70/30 for training and testing, respectively. The split was performed 10 times to create 10 outer folds. Within each of the 10 outer folds, the glmnet (Friedman et al., 2010) and glmnetUtils packages were used to perform the elastic net regression, including 10‐fold inner cross validation to determine the optimal values for alpha and lambda (hyperparameter optimisation). Using the minimum value of alpha, the model was fitted to the training data for 100 values of lambda. The resulting model was then used to predict lifespan values for the training and testing data, specifying the optimal lambda.1se (lambda “one standard error”; the largest value of lambda within one standard error of the minimum lambda value) from the previous cross validation step. Pearson's correlation coefficients between known and predicted lifespan values were calculated for both the testing and training datasets. Comparisons between the testing and training data correlations and residuals were identified using Fisher's *z* test (cocor R package) (Diedenhofen & Diedenhofen, 2016) and Students unpaired *t*‐test, respectively. The results of each of the 10 models where then bagged (bootstrap aggregated) to produce more accurate lifespan predictions (Breiman, 1996). To enable correlations between prediction error and distance from the zebrafish last common ancestor, a tree including all chordates was obtained using TimeTree (Kumar et al., 2017). The chordate tree was then subset for all fish species in our data set, and pairwise distances between zebrafish and all other species were calculated using the ape package (Paradis et al., 2004). Tree data, promoter CpG O/E and lifespan data were visualized using the ggtree package (Yu et al., 2017).

### 2.5 Gene ontology and analysis

Gene ontology (GO) enrichment was performed using gprofiler2 (an interface to the gprofiler tool g:GOSt) (Kolberg et al., 2020) specifying zebrafish as the reference organism. The analyses were performed on all promoters used to predict lifespan, divided into two groups based on the weighting of their average coefficient values (negative or positive).

## 3 RESULTS

### 3.1 Fish lifespan prediction

#### 3.1.1 Final data set

**FIGURE 1:**
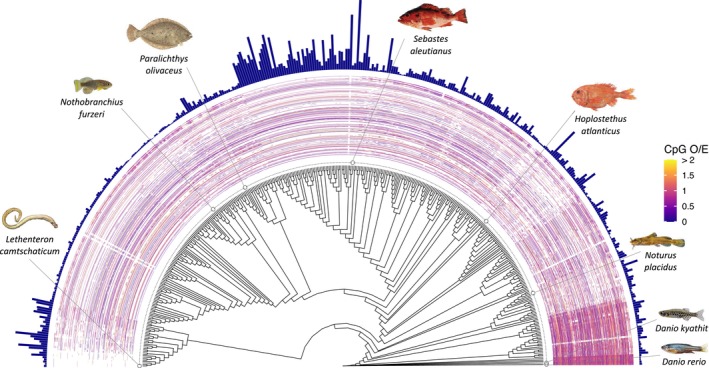
Overview of data used to build the fish genomic lifespan predictor. Each tip of the chronogram (derived from TimeTree.org) represents a single fish species, where the root species is zebrafish (*Danio rerio*). The associated CpG observed over expected ratio (O/E) in promoter regions is shown in the heatmap, where the grey colour indicates missing values (the absence of a blast hit). The known lifespan for each species, here defined as the mean of all reported lifespans, is represented by the height of the blue bars (range ≈ 1–183 years). The figure illustrates the variability in promoter coverage and fish lifespan data used to train and test the model and is labelled with eight species mentioned within the main text.

A total of 1804 reported lifespan values were obtained from six online databases, 10 published data sets and over 100 additional species‐specific publications (Figure S1, Table S1). The reported lifespans were used to calculate known lifespan estimates (i.e., the mean of the reported lifespan values for each species) for 442 fish species with publicly available genome assemblies (Figure 1, Table S1, Figure S2). The number of species per fish class was as follows: Actinopteri (*n* = 424), Cladista (*n* = 2), Chondrichthyes (*n* = 9), Cephalaspidomorphi (*n* = 0), Hyperoartia (*n* = 4), Myxini (*n* = 0) and Sarcopterygii (*n* = 3). Known lifespan values ranged from mean 0.57 (SD 0.46) years for the Turquoise killifish (*Nothobranchius furzeri*) to mean 183.33 (SD 33.57) years in the rougheye rockfish (*Sebastes aleutianus*) (Figure 1, Table S1, Figure S2). Orange roughy (*Hoplostethus atlanticus*) exhibited the greatest variance in reported lifespan values, with a mean 85.57 (SD 59.24) and a range of 10–149 years (Table S1, Figure S2).

The maximum number of blast hits to a total of 10,230 zebrafish promoter regions was 9447 in the orange finned danio (*Danio kyathit*), and the minimum eight hits in the Arctic lamprey (*Lethenteron camtschaticum*) (Figure 1). The genome assembly for the common whitefish (*Coregonus lavaretus*; GCA_905477555.1) returned zero hits, precluding lifespan prediction for this species. The *C. lavaretus* assembly is a highly incomplete (busco genome completeness score of 0%) metagenome assembled genome (MAG) from fragments obtained to assess the gut microbiota of salmonids, but not the host genome specifically (Rasmussen et al., 2021). The average hit length for the 201 bp region across all 10,230 promoters ranged from 177.11 bp in *D. kyathit* to 0.05 bp in *L. camtschaticum*. According to TimeTree, the estimated divergence time between zebrafish and orange finned danio, and zebrafish and Arctic lamprey are 16.4 million years and 599 million years, respectively. CpG O/E values within the promoter blast hits ranged from 0 to 28, with a minimum non‐zero value of 0.06 (Figure 1). The number of blast hits, blast hit length and the average CpG O/E all decreased with divergence time from zebrafish (Figure 1, Figure S3). Known lifespan increased, although the relationship was not significant (Figure 1, Figure S3).

#### 3.1.2 Model cross validation

Ten‐fold nested cross validation resulted in 10 models with lambda.1se values ranging from 1.79–4.34, where the lower penalty values were associated with lower mean squared error in the training data but larger differences in the residuals between testing and training model predictions (i.e., overfitting; Figure S4). Minimum alpha values ranging between 0.01 and 0.03, indicating that lifespan predictions with lower error are produced using a penalty ratio closer to 0 (ridge regression; L2 penalty) than 1 (lasso regression; L1 penalty) (Figure S4). The lower alpha value indicates that the lifespan model is more accurate where a larger number of features (here, promoters) are included. The number of promoters included in each model ranged from 144 to 541, and 126 promoters were represented in all 10 models (Figure S5). Despite the variance in the promoters used to predict fish lifespan, the correlations between known and predicted lifespans were consistent across models incorporating different combinations of promoters. Specifically, for all 10 models, the Pearson's correlation coefficient was greater than .7 (training: *R* = .8–.87; testing: *R* = .7–.74), the coefficient of determination was greater than .49 (training: *R* ^2^ = .63–.76; testing: *R* ^2^ = .49–.54) and the correlation *p*‐value was less than .05 (Figure S6).

#### 3.1.3 Lifespan model, prediction accuracy and variability

**FIGURE 2:**
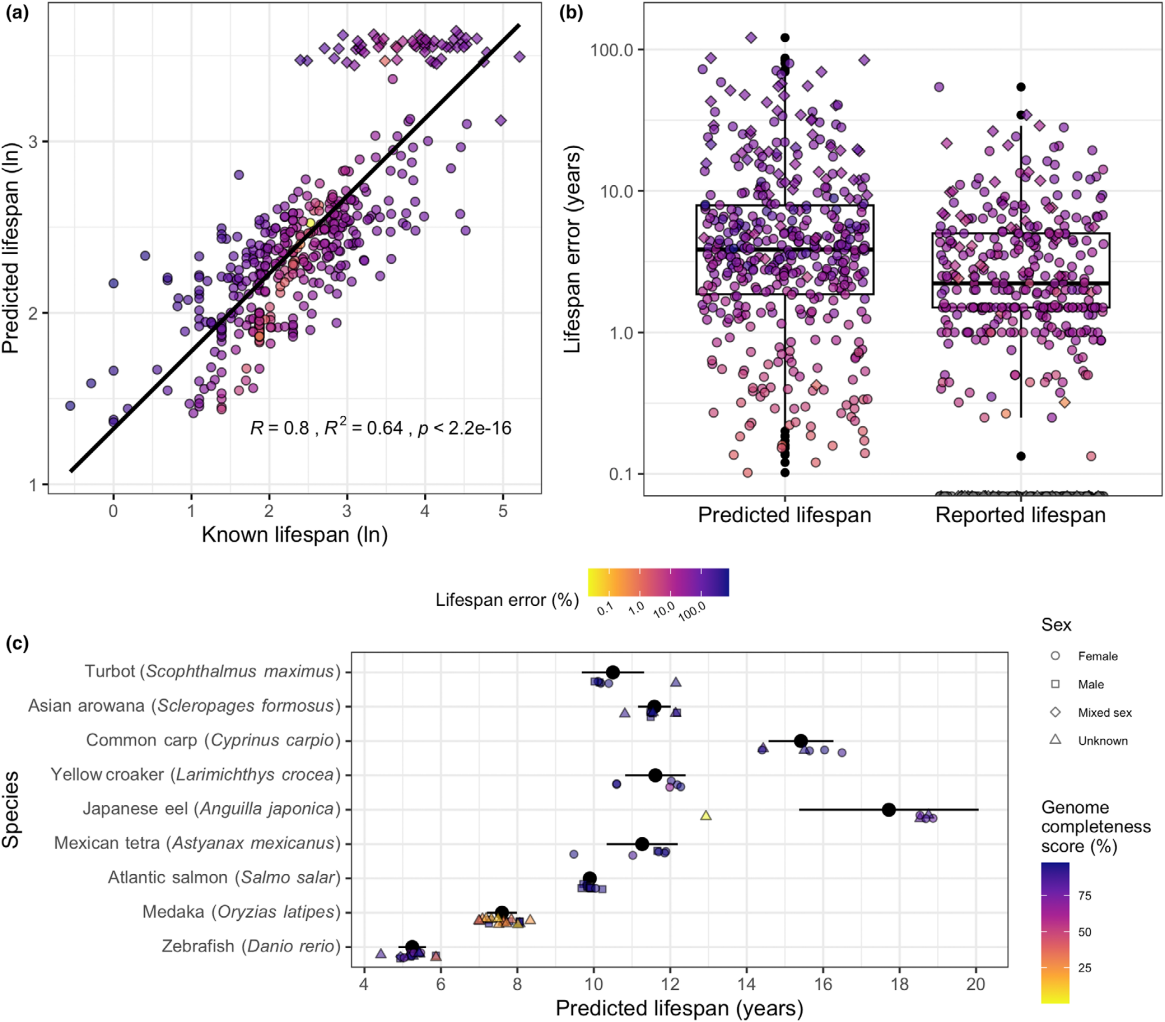
Results of the fish lifespan model. (a) Correlation between the mean value for all reported lifespans (known lifespan) and lifespan values as predicted by the model (predicted lifespan). (b) Comparison of the error in predicted and reported lifespan values. Error is calculated as the difference between the known and predicted lifespans and the known and reported lifespans, respectively. Error is presented as absolute difference (in years) on the x‐axis and relative difference (%) according to the coloured bar. Diamond‐shaped points indicate species from the *Sebastes* genus, circles indicate all others. (c) Variability in lifespan values predicted for each of nine fish species for which at least five unique genome assemblies and associated NCBI biosamples were available. Black points and bars indicate mean and standard deviation, respectively. Additional points represent lifespan predictions for each genome assembly, coloured by their busco genome completeness score and shaped according to sex, where reported.

The final model used a total of 932 promoters to predict fish lifespan with a correlation coefficient of .8 (*p* < .001), explaining 64% of the total variance between known and predicted lifespans (Figure 2a). The median relative and absolute error for all predicted lifespans were 3.81 years and 36.78%, respectively, and were approximately double the median absolute and relative error of 1.5 years and 20% for the known lifespan values (Figure 2b). The least accurate prediction in terms of relative error was for the Neosho madtom (*Notorus placidus*) with a known lifespan of 1 year, a predicted lifespan of 8.97 years and a relative error of 797.11%. The least accurate prediction in terms of absolute error was for the rougheye rockfish (*S. aleutianus*), with a known lifespan of 183.33 years, a predicted lifespan of 33.07 years and an absolute error of 150.26 years (Table S3). The most accurate prediction was for the olive flounder (*Paralichthys olivaceus*) with a known and predicted lifespan of 12.5 years, a relative error of 0.02% and absolute error of 0 years (Table S3).

Lifespan predictions produced using different genome assemblies (and associated biosamples) for a given species were highly consistent, with standard deviations of less than 1 year for all species (Figure 2c; Table S4). The sole exception was the Japanese eel (*Anguilla japonica*), for which one of the assemblies had a busco genome completeness score of 0.1% (Figure 2c, Table S5). This resulted in a lifespan prediction that was approximately 8 years less than that produced by the remaining five eel assemblies (Figure 2c, Table S5). Genome completeness score did not correlate with error in the predicted lifespans, demonstrating that the model is highly robust to low quality genome assemblies (Figure S7G). However, the very poor quality of the Japanese eel genome assembly and associated prediction suggest that a low stringency cutoff (e.g., 10% complete) would be beneficial.

#### 3.1.4 Variables associated with error in lifespan prediction

There was no correlation between relative error in the predicted lifespans and: (1) known lifespan; (2) predicted lifespan; (3) relative known lifespan error or (4) the number of reported lifespan values used to calculate known lifespan (Figure S8). However, the number of reported values resulted in a correlation coefficient with relative error of 0.08 (*p* < .1), suggesting that known lifespan estimates derived from a larger number of input values may lead to lower percent error in the predictions (Figure S8D). To further investigate this relationship, generalized linear modelling (GLM) was carried out to model percent prediction error and known lifespan, the number of known lifespan values and the interaction between the two. The GLM revealed that this trend (of more input values leading to lower prediction error) was both influential and significant, but only for shorter lived species (less than 40‐year lifespan; Table S6, Figure S9). This probably reflects a general tendency of smaller measured values to have higher relative error (e.g., Figure S8a; *p* < .1).

**FIGURE 3:**
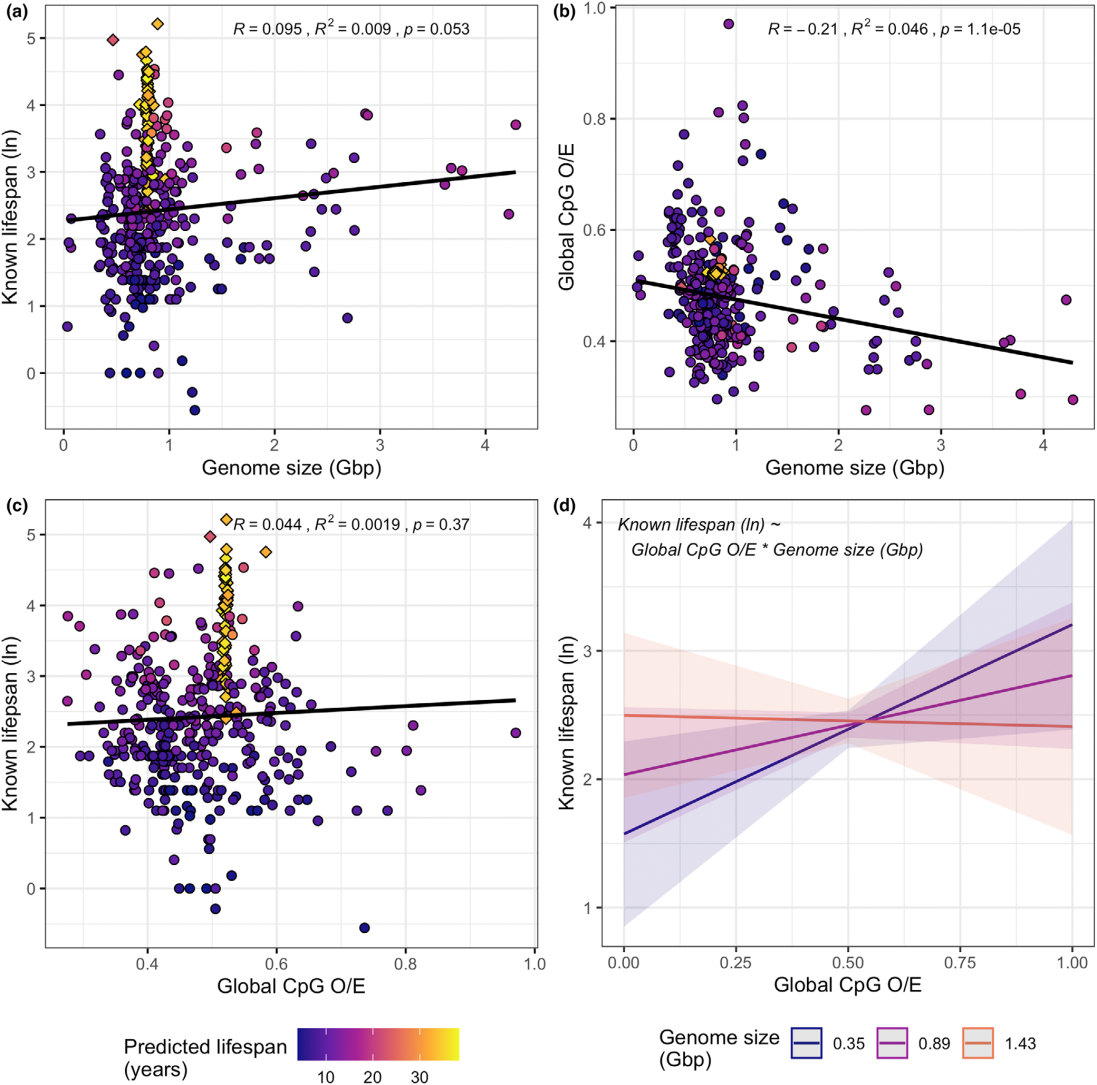
Relationships between global (genome‐wide) CpG observed/expected ratio (O/E), genome size and fish species lifespan. Pearson correlations are given for: (a) genome size in gigabase pairs (Gbp) and known lifespan; (b) genome size and global CpG O/E and; (c) global CpG O/E and known lifespan. Diamond‐shaped points indicate species from the *Sebastes* genus, circles indicate all other species. (d) Shows the interaction between global CpG O/E and genome size on known lifespan as predicted by generalized linear modelling (formula shown in the top left), where shaded areas indicate confidence intervals. The figure indicates that the relationship between known lifespan and CpG O/E is dependent on genome size.

No significant correlations were identified between the relative error for predicted lifespans and: (1) the total number of blast hits; (2) mean blast hit length; (3) mean sequence identity; (4) genome assembly completeness (busco completeness score) or (5) divergence time from zebrafish (Figure S7). However, the variance in divergence times produced by TimeTree was limited, where the pairwise distances were uniform for 75% of species (Figure S10). Nonetheless, negative trends for hit number and hit length suggests that decreases in promoter sequence information used by the lifespan model led to decreases in prediction accuracy, although the variance was large (Figure S7). The range of predicted lifespans was smaller than known lifespan range, most obviously in species of the *Sebastes* genus (Figure 2a). In general, CpG O/E values were less variable among *Sebastes* spp. compared to fish in other genera, although known lifespans varied considerably (e.g., Figure 3a, Figure S11). Invariable CpG O/E values may have led to an inability of the model to accurately predict the highly variable lifespans of fish from this group. This is difficult to measure statistically due to the over representation of *Sebastes* species in the data set (57 *Sebastes* species compared to a mean of 1.56 for all other genera).

### 3.2 Model composition

#### 3.2.1 Promoter correlations and model weighting

**FIGURE 4:**
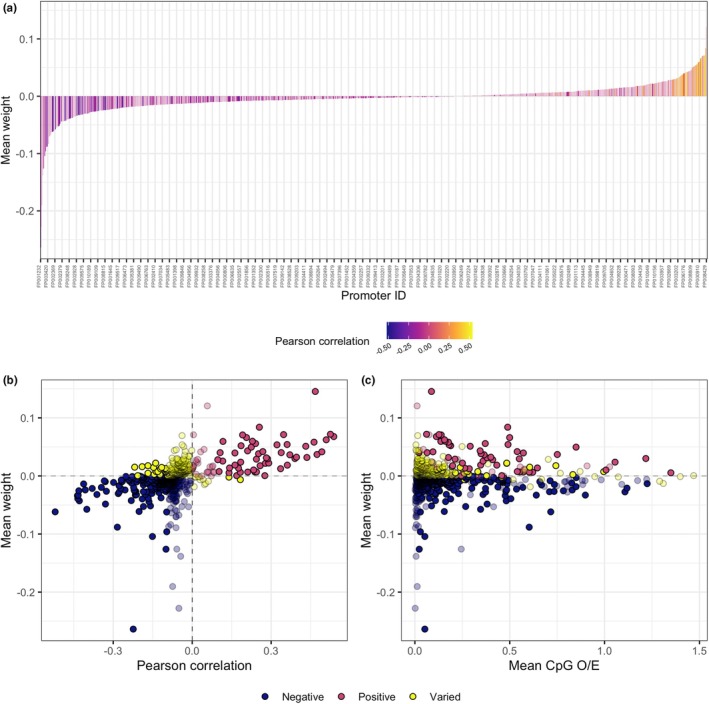
Overview of relationships between modelled promoters and fish species lifespan. (a) Mean weighting for each promoter in the lifespan prediction model ordered by magnitude and directionality (large negative to large positive). Bars are coloured by the Pearson correlation coefficient between known lifespan and observed over expected ratio (CpG O/E) for each promoter. Only every tenth promoter is labelled on this x axis (total *n* = 932). (b) Pearson correlation coefficients for each promoter compared to mean weighting in the lifespan model. (c) Mean CpG O/E for each promoter compared to mean weighting in the lifespan model. Colours indicate if both the correlation and model weighting were negative, positive, or varied between the two as per the legend. Transparent bars and points indicate that the Pearson correlation between promoter CpG O/E and lifespan was not significant.

Cytosine‐phosphate‐guanine O/E was negatively associated with lifespan for more than 60% of promoters in the model (Figure 4). Specifically, of a total of 932 promoters in the lifespan model, 582 were negatively weighted, and 350 were positively weighted (Figure 4). These results were consistent with Pearson correlations for negatively weighted promoters, where 570 promoters were negatively correlated with lifespan, and only 12 were positively correlated (Figure 4b). The results were more varied for promoters positively weighted in the lifespan model, where 274 had negative Pearson correlations and 76 had positive Pearson correlations (Figure 4b). The promoter weights (coefficients) for each of the 10 bagged models produced during outer‐fold cross validation are presented in Table S7.

#### 3.2.2 Promoter CpG observed over expected ratios

Cytosine‐phosphate‐guanine O/E was 0 for 96% of all promoters in the complete data set and 82% of promoters in the model. Mean CpG O/E values were significantly higher in the selected promoters compared to those not selected by the model (Figure S12a). However, when zero values derived from the absence of a blast hit were removed from the data set, the pattern was reversed (Figure S12b). These results indicate that the model selects for promoters with non‐zero CpG O/E values, but beyond this does not select for larger CpG O/E values. The promoter weights were more variable and of larger magnitude for smaller values of mean promoter CpG O/E; however, the data was skewed toward smaller CpG O/E values (i.e., CpG O/E < 0.25; Figure 4c).

### 3.3 Additional model testing

Model testing for different data subsets, known lifespan measures (mean or median) and promoter lengths revealed that the final model, using the full dataset, the mean of all reported lifespans and the core promoter (−100 to +100 bp) produced the most accurate lifespan predictions. Data subsets: (1) excluding all FishBase data (Froese & Pauly, 2010); (2) excluding any data with no primary source information available; and (3) including only AnAge data (de Magalhães & Costa, 2009) all produced higher prediction error and lower *R* ^2^ values compared to using the full dataset (Figure S13). When employing the median of all reported lifespans as the known lifespan value, the correlation between known and predicted lifespan was marginally worse than when using the mean (Figure S14a,b). However, because the mean and median of the reported lifespans are very similar measures, the results were very similar (Figure S14c). All promoter lengths tested (−100 to +100, −499 to +100 bp and −500 to +1500 bp) gave reasonable prediction accuracy (known~ predicted *R* ^2^ > .49), demonstrating the robustness of the lifespan model to different genomic regions surrounding the TSS (Figure S15). Larger promoter regions offered a better fit for the training data; however, this was not the case for the testing data suggesting some degree of overfitting (Figure S15). These differences were minimized when using the core promoter region (−100 to +100 bp) (Figure S15) and as such, this region was employed for the final lifespan model.

### 3.4 Functional analysis

**FIGURE 5:**
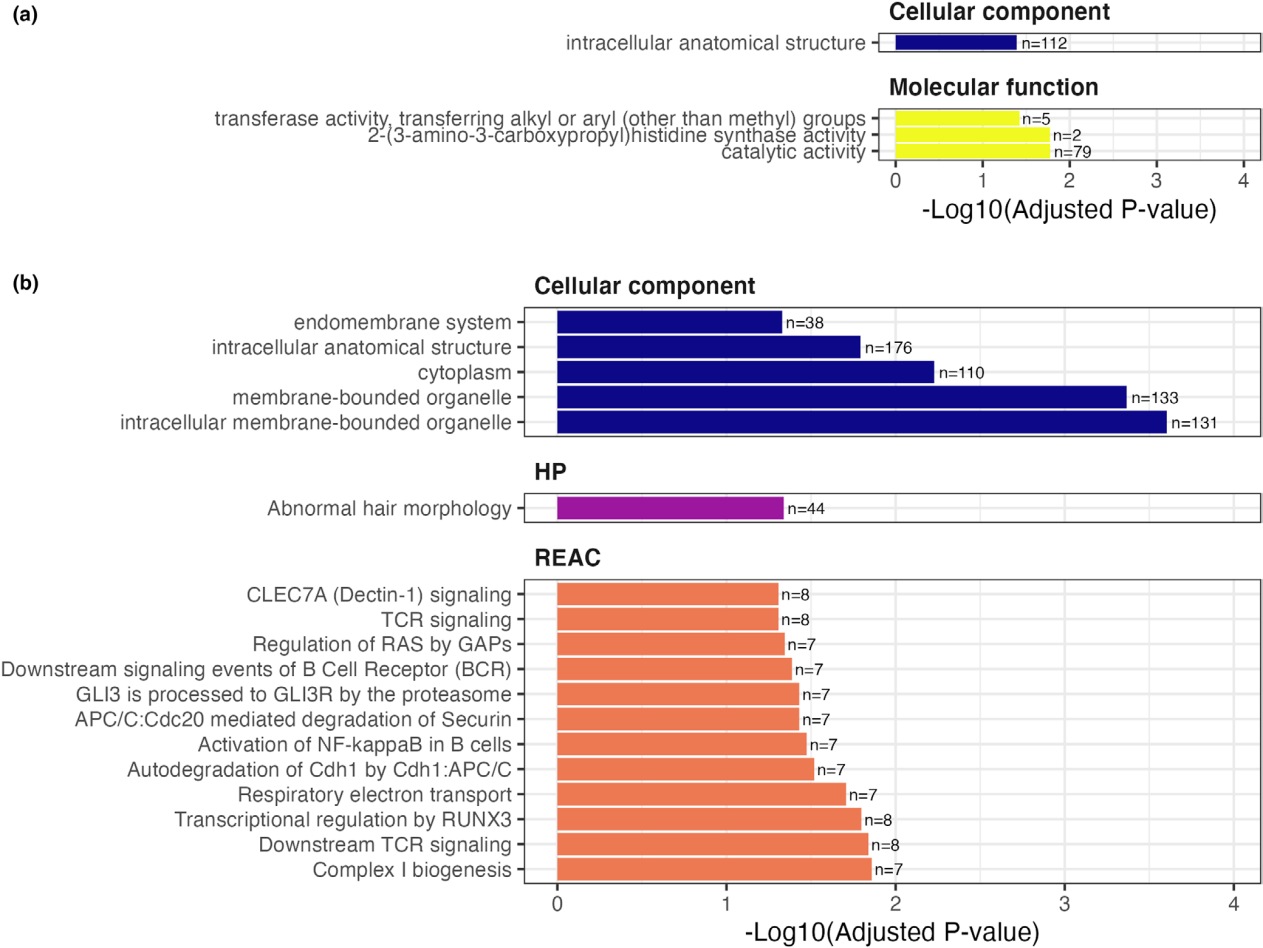
Functional enrichment analysis showing biological functions and pathways over‐represented in the fish lifespan model promoter associated genes for: (a) promoters positively weighted in the model and (b) promoters negatively weighted in the model. HP indicates functions derived from the Human Protein Atlas, and REAC from the Reactome database. A table of these results can be found in Table S8.

Functional analysis revealed enrichment for genes associated with several GO terms, Reactome pathways and tissue specificity from the Human Protein Atlas (Figure 5). Promoters positively weighted in the lifespan model were enriched for genes associated with intracellular anatomical structures and catalytic activity (Figure 5a). Negatively weighted promoters were enriched for genes with functions largely related to intracellular components, including those involved in cellular transport (Figure 5b). Negatively weighted genes were also enriched for various biological signalling pathways from the Reactome data base. These include five with roles in immune system functioning (downstream signalling events of B cell receptor (BCR), CLEC7A (Dectin‐1) signalling, TCR signalling, downstream TCR signalling and activation of NF‐kappaB in B cells), two in signal transduction (GLI3 is processed to GLI3R by the proteasome, regulation of RAS by GAPs), two in metabolism (respiratory electron transport, complex I biogenesis), two in cell cycling (autodegeneration of Cdh1 by Cdh1:APC/C, APC/C:Cdc20 mediated degradation of Securin) and one in gene expression (Transcriptional regulation by RUNX3; Figure 5b).

### 3.5 Global trends

No significant Pearson correlation between global CpG O/E and species known lifespan or genome size was observed; however, genome size was negatively correlated with global CpG O/E (Figure 3). A subsequent GLM revealed the relationship between CpG O/E and lifespan is apparent (despite the absence of a Pearson correlation) but is influenced by the interaction between global CpG O/E and genome size. More specifically, while known lifespan increases with global CpG O/E, this relationship is reduced, and even reversed as genome size increases (Figure 3d, Table S9).

## 4 DISCUSSION

Using publicly available data from 442 fish species comprising five vertebrate classes, we developed a model to predict species maximum lifespan from genomic CpG density alone. The accuracy of the fish lifespan predictions was consistent across genome assemblies of different samples of the same species, indicating that the analysis of a single individual is sufficient to predict a species' lifespan using this method. We anticipate this novel approach having immediate utility in any fishery management case where lifespan approximation by other means is impracticable, and here identify areas for future research that may improve the predictive power of the model for broader application.

### 4.1 Robustness, accuracy, and potential application of genomic lifespan prediction

The fish lifespan model demonstrates that there is a strong association between genomic CpG density and lifespan. Based on this association, the model is robust to sequence differences between zebrafish promoters and orthologous promoters in distantly related species, as well as differences in genome assembly completeness. The resulting predictions had approximately double the error of the reported values of lifespan, which require far more intensive research efforts to obtain. To predict lifespan using this method, the genome sequence of just a single individual (no repeated sampling) is required. This involves the acquisition of a small piece of tissue (e.g., a fin clip), genome sequencing and assembly followed by downstream bioinformatic analysis. Contig‐level assemblies for genomes up to 1 Gbp in size (i.e., most fish) can be produced for less than $5000 USD and in under 2 weeks (R. Huerlimann, personal communication). If a genomic assembly for the species is already available, model predictions can be generated immediately and with no associated consumable expenses. At present, lifespan estimation involves either tagging and repeated sampling in the field to determine maximum observed age (de Magalhães & Costa, 2009), modelling the maximum based on trends in survivorship with age (Mayne et al., 2020) or estimations based on maximum length (Taylor, 1958). The cost and time involved in housing animals in aquaria or monitoring enough individuals to confidently identify or calculate maximum age using current methods probably far exceeds what is required for genomic lifespan prediction.

### 4.2 Molecular predictors of lifespan

In addition to providing lifespan predictions, the model may provide insight into the molecular biology of fish lifespan. For example, it has been suggested that the association between genomic CpG density and lifespan is due to a protective effect of increased CpG density against age‐related epigenomic changes (Bertucci & Parrott, 2020). Previous results in mammals showed that CpG density is positively associated with lifespan in 94% of promoters (McLain & Faulk, 2018), providing strong support for this theory. However, the vertebrate model showed this positive association was only present for 62% of modelled promoters (Mayne et al., 2019) and here we observed positive associations in just 38%. These results highlight that differences in CpG density are important for predicting lifespan, rather than simply increases, as previously hypothesised. This is evident in mammals and other vertebrates, but is particularly pronounced in fish.

Previous functional analyses of lifespan‐related promoters in CpG density models have been unable to identify any significantly enriched gene functions (Mayne et al., 2019; McLain & Faulk, 2018). However, analysis of the lifespan‐associated genes here revealed functions related to intracellular components, transport and immune functioning pathways. Specifically, we identified a number of pathway components related to T and B cell functioning as well as NF‐KB signalling pathways, all of which are of central importance in immune functioning. Transcriptional regulation by RUNX3 was also identified; a gene that functions in the suppression of tumours (Spender et al., 2005). Collectively, these immune system components are protective against toxins, infection, and cancer and thus are highly likely to influence longevity (Baltimore, 2009; Clark & Ledbetter, 1994). These results are consistent with epigenetic age predictors, which commonly select for genomic regions associated with immune function (Liu et al., 2020).

We also observed enrichment for specific signal transduction pathway elements, with many involved in Hedgehog repression and RAF/MAP kinase pathways, which regulate programmed cell differentiation and aspects of immune functioning (Briscoe & Thérond, 2013; Crompton et al., 2007; Krens et al., 2006). Interestingly, the analysis revealed enrichment for 44 genes associated with abnormal hair formation in humans. Due to the presence of many shared signalling pathways, Actinopterygian scales are thought to be evolutionary precursors to mammalian hair, which is known to degenerate with increasing age (Sharpe, 2001). Fish also have hair cells in their lateral line for sensing prey as well as in their ear canals for sensing barometric pressure (Bleckmann, 2006; Heupel et al., 2003). Promoters for genes that are important for species survival may have been altered in different lineages under varying selection pressures, leading to lifespan changes among fish species.

We observed no Pearson correlation between global CpG O/E and lifespan. This provides some support for the hypothesis that age‐related changes in DNA methylation in promoter regions specifically (as opposed across the genome more generally) are strongly associated with lifespan (McLain & Faulk, 2018). In contrast, when genome size and the interaction between genome size and CpG O/E were controlled for, we observed a positive relationship between global CpG O/E and lifespan for small genomes and a negative relationship for large genomes. We also observed a significant negative Pearson correlation between genome size and CpG O/E. This is consistent with previous reports that high levels of DNA methylation (and therefore low CpG O/E) lead to increases in genome size via the suppression of transposable element (TE) activity (Zhou et al., 2020). The differing relationship between global CpG O/E and lifespan for larger genomes may therefore be related to increased TE load. However, as this was not the focus of the work, the present results are inconclusive. The relationship between global CpG O/E, genome size, and how it relates to species lifespan warrants further investigation.

### 4.3 Limitations and future directions

Despite the broad applicability and predictive power of the fish lifespan model, variable levels of prediction accuracy may limit its application in its present form. The accuracy of machine learning models, including elastic net regression, is substantially impaired by poor quality training data (e.g., incorrect, inconsistent, or missing values) (Sun et al., 2017). In many cases, increasing sample size and using techniques such as cross validation and bagging as applied here will reduce the effects of outliers and increase model accuracy (Gudivada et al., 2017). Our model predictions would be further improved if the quality of the training data (here, the known lifespan values) were increased. Maximum age and therefore lifespan values are difficult to determine for many fish species. The most common aging technique in bony fish, otolith aging, is subject to observation error and is especially difficult to perform for long‐lived species. For example, reported orange roughy lifespan estimates range from 10 to 230 years, and despite extensive investigation the true value is still disputed (Andrews et al., 2009; Horn & Maolagáin, 2019). For cartilaginous fish (sharks and rays), lifespan estimation is particularly difficult because a reliable method for aging is yet to be established (Burke et al., 2020). At present, the fish lifespan model relies upon existing lifespan data for training and validation. As such, improvements in the accuracy of training data would probably improve the accuracy of the model's predictions. There is little research on how to measure data quality for robust machine learning model development, although software tools for data quality control are emerging in different fields (Ehrlinger et al., 2019).

The lifespan model training data also suffers inconsistency in taxonomic coverage. For example, the over representation of *Sebastes* species (*n* = 57), or the under‐representation of chondrichthyans (*n* = 9). To overcome this, the model could be recalibrated with additional fish genome sequences with broad taxonomic coverage as they are released from individual sequencing projects, or by collaborative efforts such as Beijing Genome Institute's Fish10K (Fan et al., 2020). Finally, a lack of sequence similarity between the target species and zebrafish resulted in reduced length or completely absent BLAST hits (i.e., a large amount of missing data). While we opted to use fish‐specific reference sequences and did not observe any bias toward higher prediction error in more divergent species, the model primarily selected promoters with nonzero values. Thus, any model using the same sequence similarity approach is likely to suffer from some degree of bias in divergent species (e.g., Mayne et al., 2019; McLain & Faulk, 2018). An alternative to using gene promoters as reference sequences may be to analyse genomic regions that can be identified by location. For example, DNA methylation in first introns and exons is highly correlated with gene expression (Anastasiadi et al., 2018; Brenet et al., 2011). However, this approach would require comparable genome annotations and would be computationally expensive to execute.

The most immediate application for the lifespan predictions is likely for the estimation of natural mortality for use in fisheries stock assessments. Lifespan (*t*_max_) based estimators consistently perform better than other methods for calculating natural mortality; one of the most widely used and difficult to estimate stock assessment parameters (Then et al., 2015). A primary advantage of both lifespan‐based estimators of mortality and the lifespan predictor presented here is the ability to provide rapid and cost‐effective analyses. The provision of this data can assist in overcoming deficiencies in expertise and expenses required to undertake formal stock assessments (approximately $50,000 USD per species) (Pauly et al., 2013). The accuracy and precision of parameter estimates varies markedly between assessments, but error rates of 10% are reported as optimal and 30% as acceptable (Goodyear, 1995; Kritzer et al., 2001). With an error rate of 37%, the model in its present form is likely to be most applicable for data limited or newly targeted fisheries, data deficient species under significant threat, and in any case where lifespan approximation by other means is impracticable.

## 5 CONCLUSION

We derived a model that predicts lifespan for any fish species from the genomic CpG density of a single individual. The model is highly robust to variation in genome quality and is applicable to all classes of fish; a taxonomically diverse and highly specious group of marked ecological and economic importance. The predictions are likely to be of use for both commercially valuable and highly vulnerable species, as lifespan enables approximation of natural mortality and rate of population increase (Dureuil & Froese, 2021; Liu et al., 2015). The work demonstrates the remarkable power of genomic CpG density alone to predict fish lifespan, and the predictive capacity of the model is likely to improve as the quantity and quality of available training data increases. Fish lifespan prediction is a significant problem for many species, and the value of estimating this fundamental life history parameter has driven interest in developing unconventional lifespan measurement technologies (Choat, 2021). We envisage the utility of our novel approach to estimate this central life history trait is likely to be far reaching, with both commercial and environmental impacts.

## AUTHOR CONTRIBUTIONS

Alyssa Budd assisted in designing the research, performed the research, analysed, and interpreted the data, and wrote the manuscript. Benjamin Mayne conceptualized and designed the research, assisted in analysing and interpreting the data and edited the manuscript. Oliver Berry and Simon Jarman conceptualized and designed the research, assisted in interpreting the data and edited the manuscript.

## CONFLICT OF INTEREST STATEMENT

The authors declare that they have no known conflicts of interest that could have influenced the work reported in this article.

## BENEFIT SHARING STATEMENT

A research collaboration was developed with scientist from the CSIRO and the University of Western Australia, and all collaborators are included as co‐authors. The preliminary results of the research have been shared to relevant government departments and universities within Australia. The results are relevant to the conservation and sustainable utilization of biological diversity.

## Supporting information


Figures S1‐S15.

Tables S1‐S9.

## Data Availability

Genomic data was downloaded from the NCBI genomes database using the accession numbers provided in Tables S2 and S4. Known lifespan data and metadata are included in Table S1. Lifespan predictions can be found in Table S3. All other data and code are available at https://github.com/dr‐budd/fish_life.
