# Supplementary material for: Fish species lifespan prediction from promoter cytosine‐phosphate‐guanine density
Source: Mol Ecol Resour. 2023 Mar 19;25(5):e13774. doi: 10.1111/1755-0998.13774 (PMC12142718; doi:10.1111/1755-0998.13774)
Supplement: Supplementary file 1 — Figures S1‐S15. [file MEN-25-e13774-s002.pdf]

MOLECULAR ECOLOGY

Supplemental Information for:

Fish species lifespan prediction from promoter CpG density

Alyssa M. Budd, Benjamin Mayne, Oliver Berry, and Simon Jarman

Table of Contents:

Figures	
**Figure S1. Overview of the fish lifespan data set.**	Page 2
**Figure S2. Distribution of lifespan values for species used in the fish lifespan model.**	Page 3
**Figure S3. Preliminary investigation of the effects of using zebrafish (*Danio rerio*) gene promoters as reference sequences in the fish lifespan model.**	Page 4
**Figure S4. Outer cross validation results for the fish lifespan model.**	Page 5
**Figure S5. Variation in feature selection among the 10 lifespan prediction models resulting from 10-fold nested cross-validation.**	Page 6
**Figure S6. Correlations between known and predicted lifespans for the training (left column) and testing (right column) data sets.**	Page 7
**Figure S7. Investigation of potential correlates and relative prediction error of the fish lifespan model.**	Page 8
**Figure S8. Relationships between known lifespan metrics (x-axes) and the relative error in predicted lifespan values (y-axes).**	Page 9
**Figure S9. Predicted values of relative error in the predicted lifespans based on the total number of reported lifespans resulting from generalised linear modelling.**	Page 10
**Figure S10. TimeTree for all fish species with lifespan and genomic information present in our dataset.**	Page 11
**Figure S11. Lack of variation in *Sebastes* promoter CpG content.**	Page 12
**Figure S12. Mean CpG observed over expected ratio (CpG O/E) for each of the 10,230 zebrafish (*Danio rerio*) promoter regions used to build the fish lifespan predictor**	Page 13
**Figure S13. Comparison of correlations and error rates for the fish lifespan prediction model when built using different reported lifespan data subsets.**	Page 14
**Figure S14. Comparison of model correlations between known and predicted lifespans under different measures of known lifespan (mean and median).**	Page 15
**Figure S15. Comparison of 10-fold outer cross validation results for the fish lifespan model using different three promoter lengths.**	Page 16

NB: Supplementary tables are presented in a separate spreadsheet document.

MOLECULAR ECOLOGY

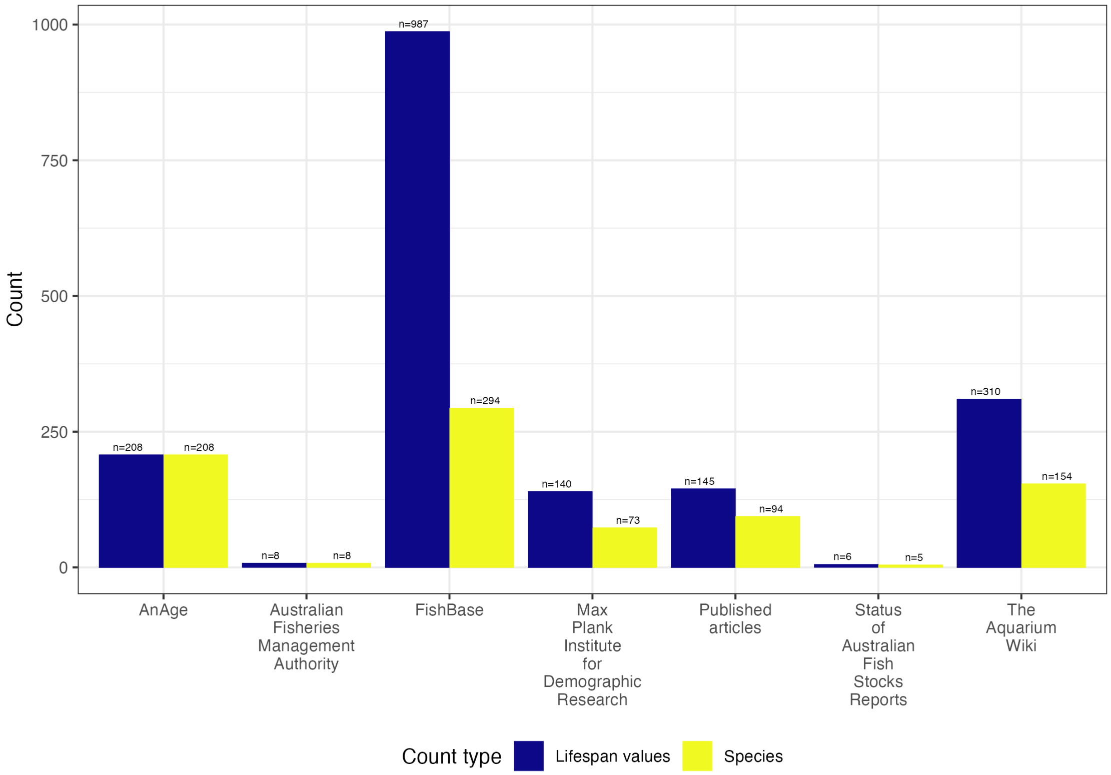

Figure S1. Overview of the fish lifespan data set. Plot shows the number of lifespan values (blue) and unique species (yellow) obtained from each source for fish species used in the present study. A table of these values is presented in Table S1.

MOLECULAR ECOLOGY

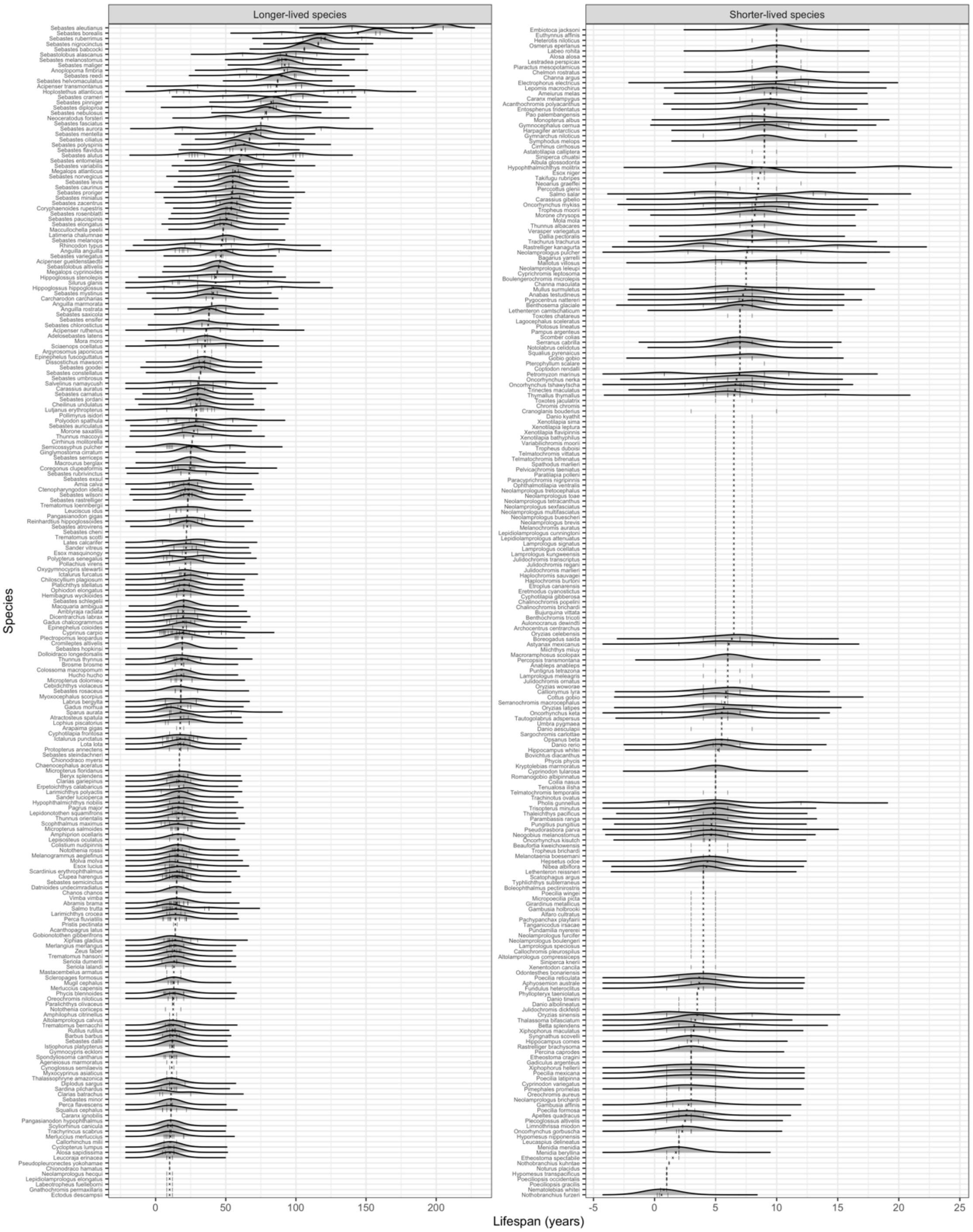

Figure S2. Distribution of lifespan values for species used in the fish lifespan model. Lifespan values were obtained from the literature and online databases, and only those with publicly available genome sequences were included in the dataset. Distribution data are presented as a ridge plot, where a line is only drawn for species with greater than two reported lifespan values. The mean and reported lifespans are additionally shown as ‘x’ and ‘|’ points, respectively. The mean was used as the ‘known’ lifespan value in all subsequent analyses.

MOLECULAR ECOLOGY

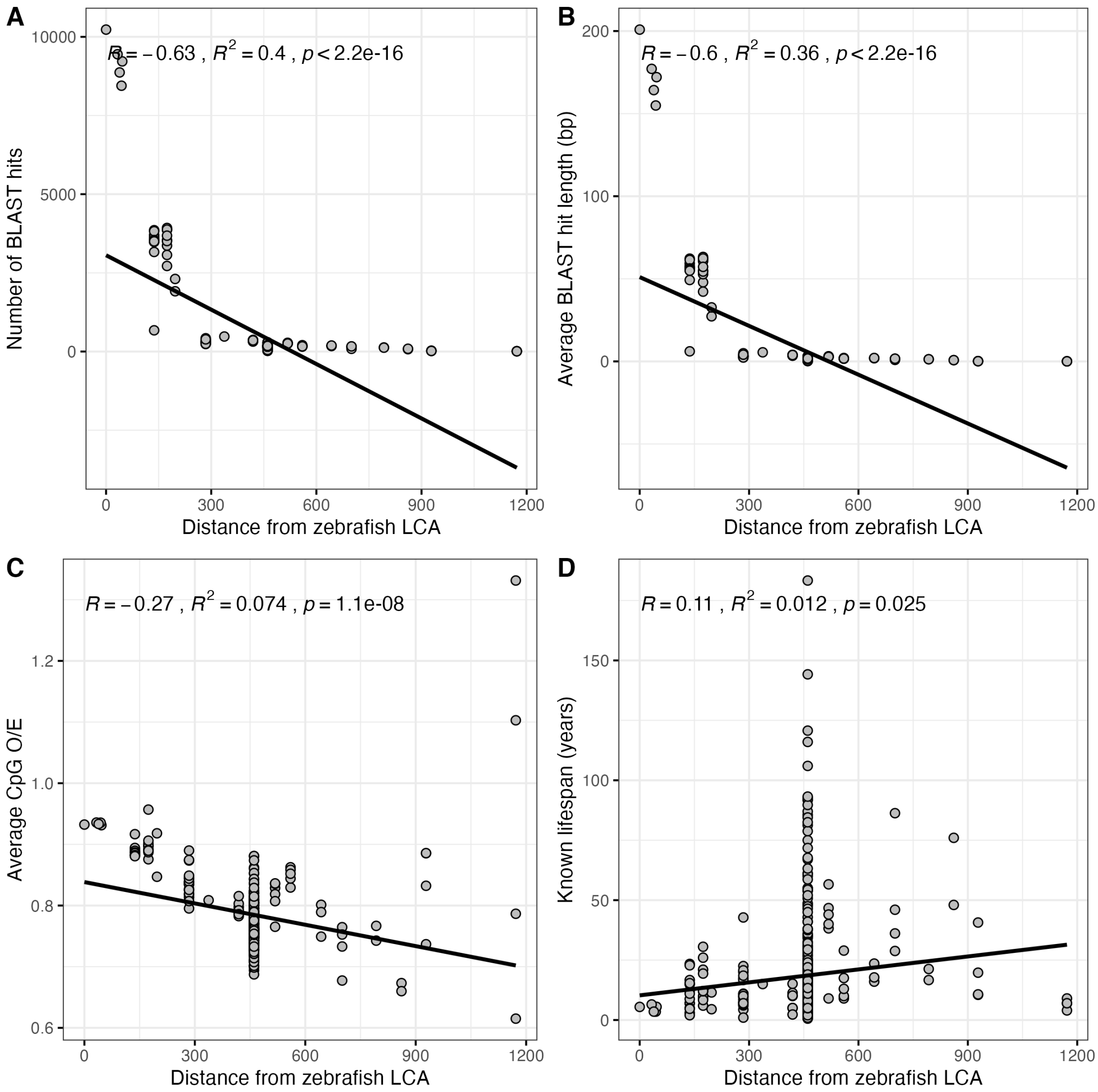

Figure S3. Preliminary investigation of the effects of using zebrafish (*Danio rerio*) gene promoters as reference sequences in the fish lifespan model. Pearson correlation for **A.** number of BLAST hits; **B.** BLAST hit length in base pairs (bp); **C.** the average CpG observed over expected ratio (O/E) or **D.** known lifespan and the distance from species last common ancestor (LCA) and zebrafish.

MOLECULAR ECOLOGY

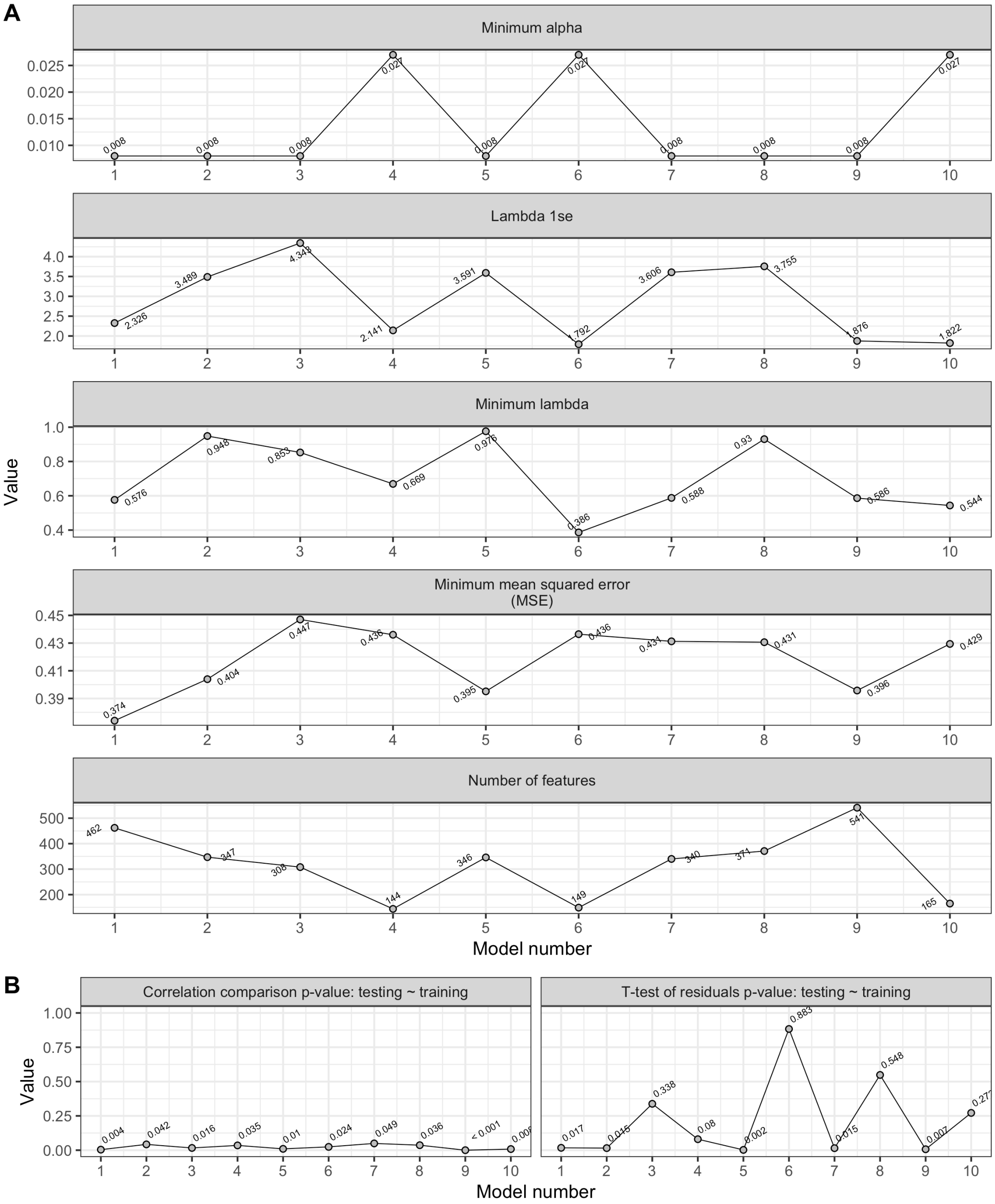

Figure S4. Outer cross validation results for the fish lifespan model. **A.** Parameters for the best performing (lowest error) lifespan models resulting from 10-fold inner cross validation for tuning of alpha and lambda. The x-axis corresponds to the model number, where the training data are derived from separate data partitions in the outer cross validation loop. **B.** Results of significance tests for the difference between correlation coefficients (left) and residuals (right) of known vs predicted lifespans for the testing and training data for each of the ten models produced during outer cross validation.

MOLECULAR ECOLOGY

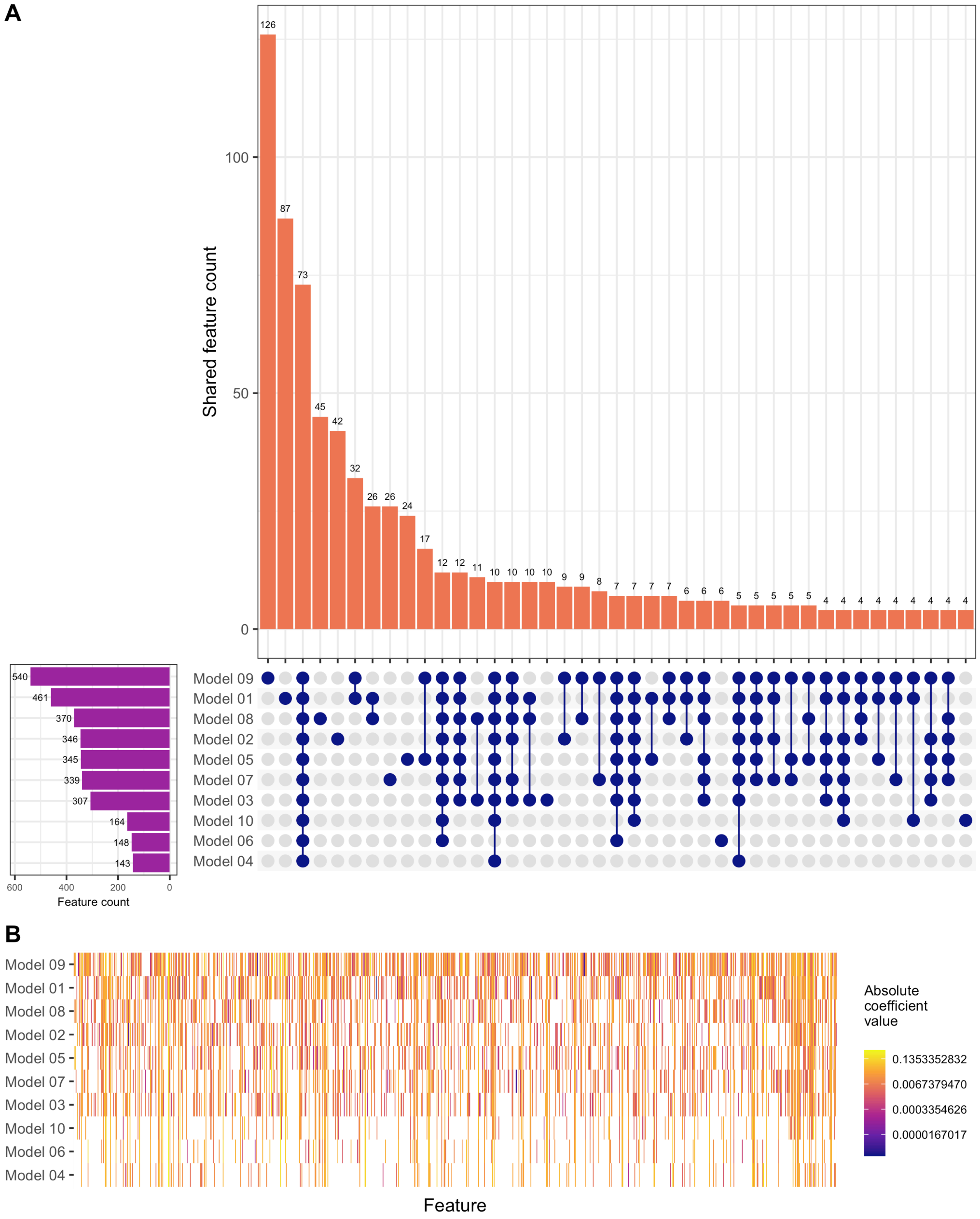

Figure S5. Variation in feature selection among the 10 lifespan prediction models resulting from 10-fold nested cross-validation. **A.** Upset plot showing which features (gene promoters) are shared between models. The purple bars indicate the total number of features included in each model, the blue matrix indicates which of the 10 models are represented by the orange bars directly above, which give the number of promoters these models share (equivalent to the overlapping components of a Venn diagram). **B.** Heatmap showing the absolute feature coefficient values among the 10 models.

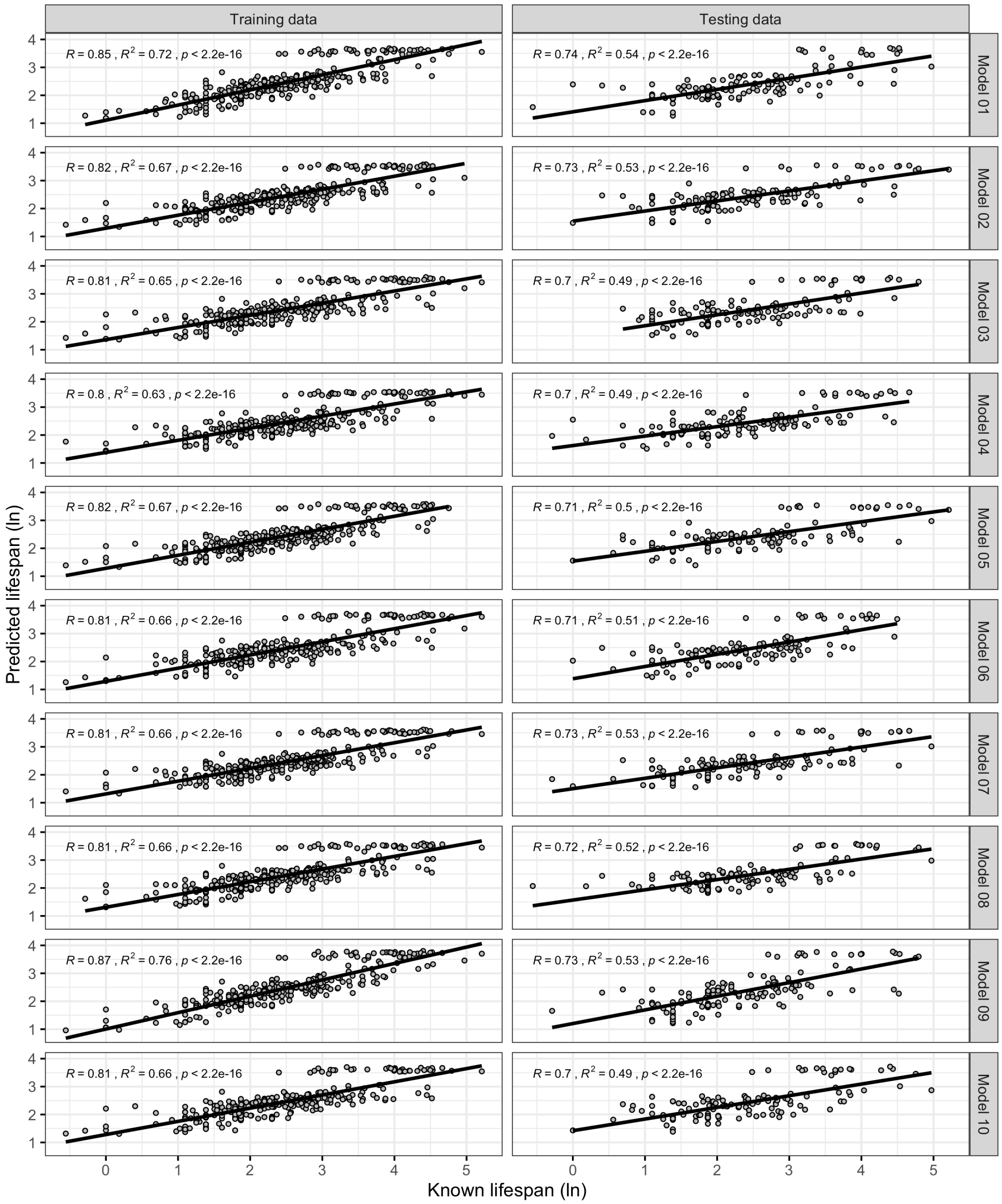

Figure S6. Correlations between known and predicted lifespans for the training (left column) and testing (right column) data sets. Each model (row) is derived from a separate testing-training data split subject to 10-fold inner cross validation.

MOLECULAR ECOLOGY

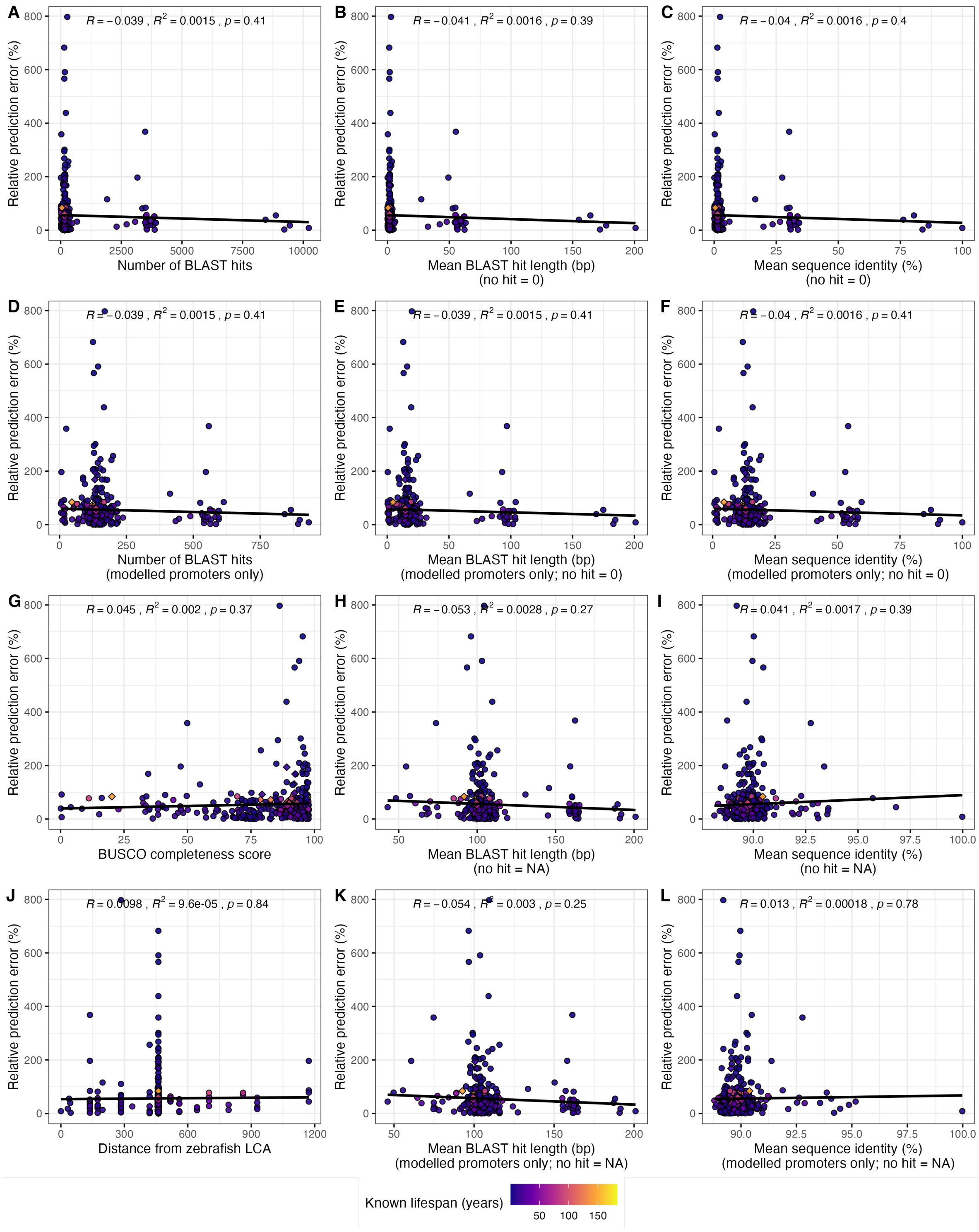

Figure S7. Investigation of potential correlates and relative prediction error of the fish lifespan model. Pearson correlations between the zebrafish (*Danio rerio*) promoter BLAST hit metrics (x-axes) and the relative error in predicted lifespan values (y-axes).

MOLECULAR ECOLOGY

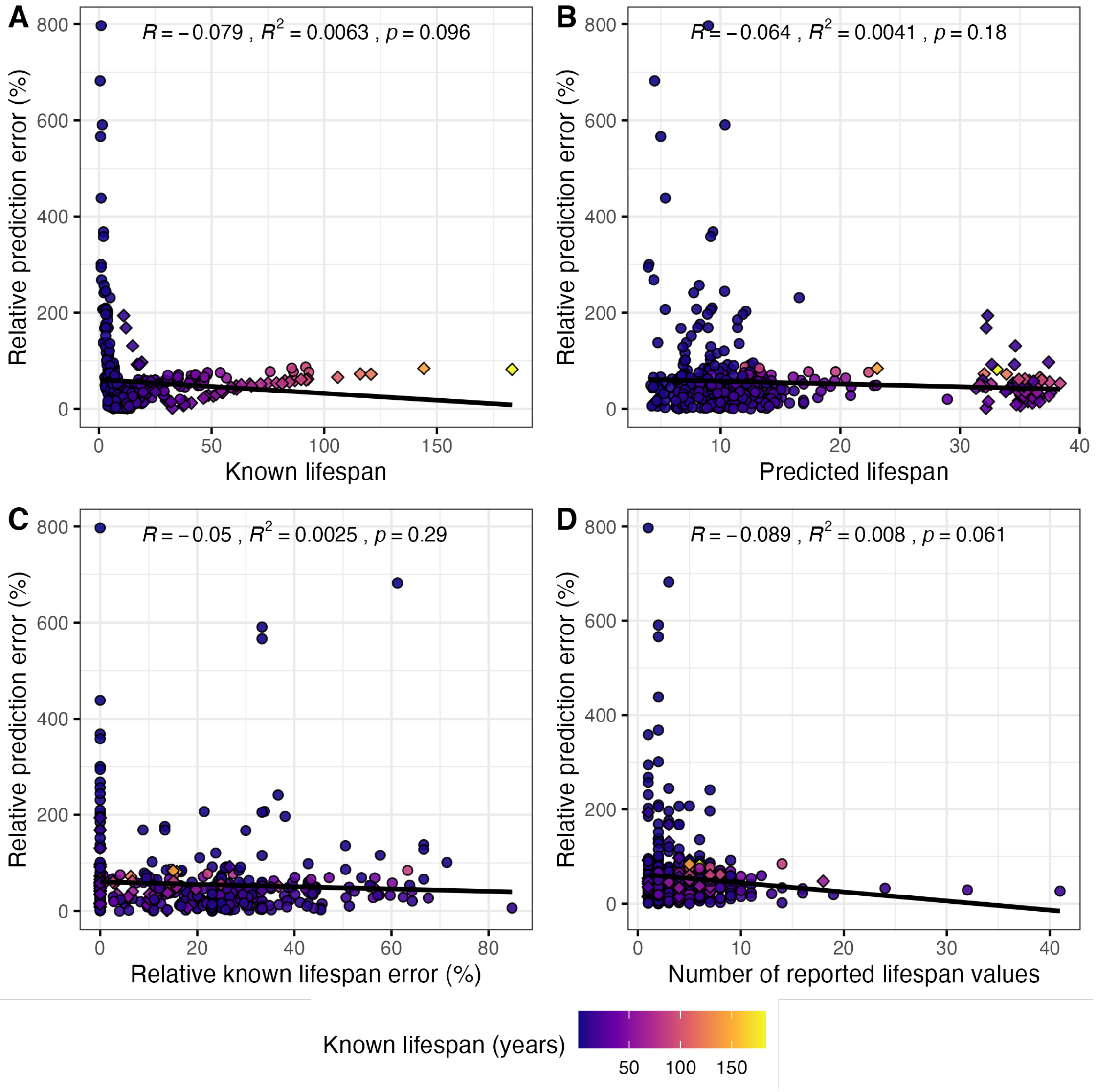

Figure S8. Relationships between known lifespan metrics (x-axes) and the relative error in predicted lifespan values (y-axes). Plots show Pearson correlations for relative error and: **A.** Known lifespan (the mean of all reported lifespan values); **B.** Predicted lifespan (the value predicted by the lifespan model); **C.** Relative error in the known lifespans and **D.** The total number of reported lifespan values in the database.

MOLECULAR ECOLOGY

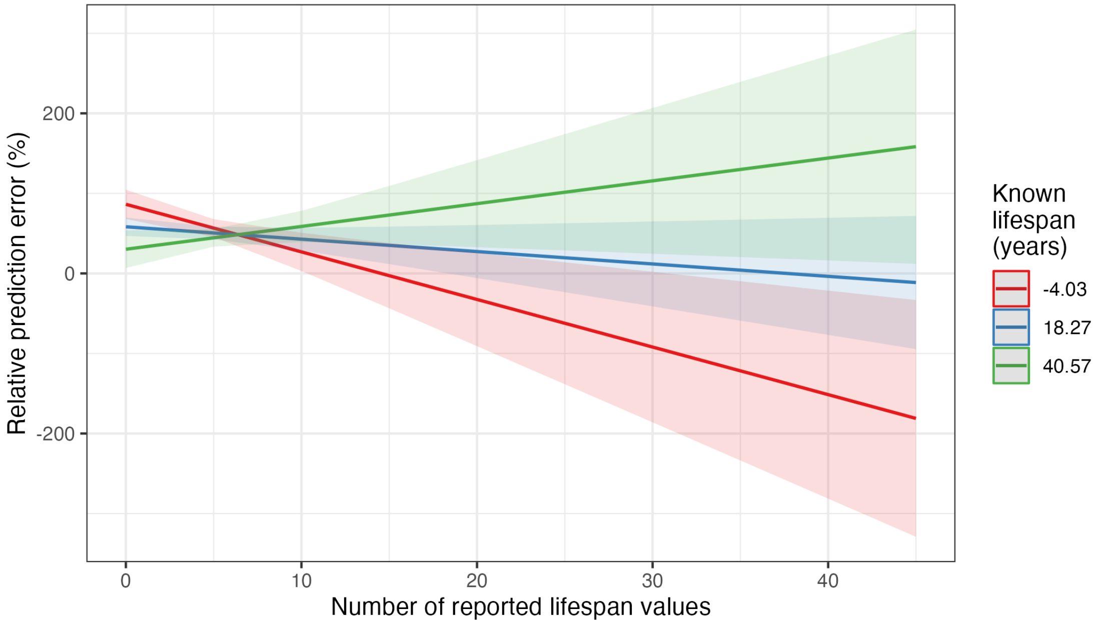

Figure S9. Predicted values (estimated marginal means) of relative error in the predicted lifespans (y-axis) based on the total number of reported lifespans in the database (x-axis), at three levels of known lifespan (the mean of all reported lifespan values) resulting from generalised linear modelling. The predictions indicate that the relative prediction error decreases with the number of reported lifespan values, but only for shorter lived species.

MOLECULAR ECOLOGY

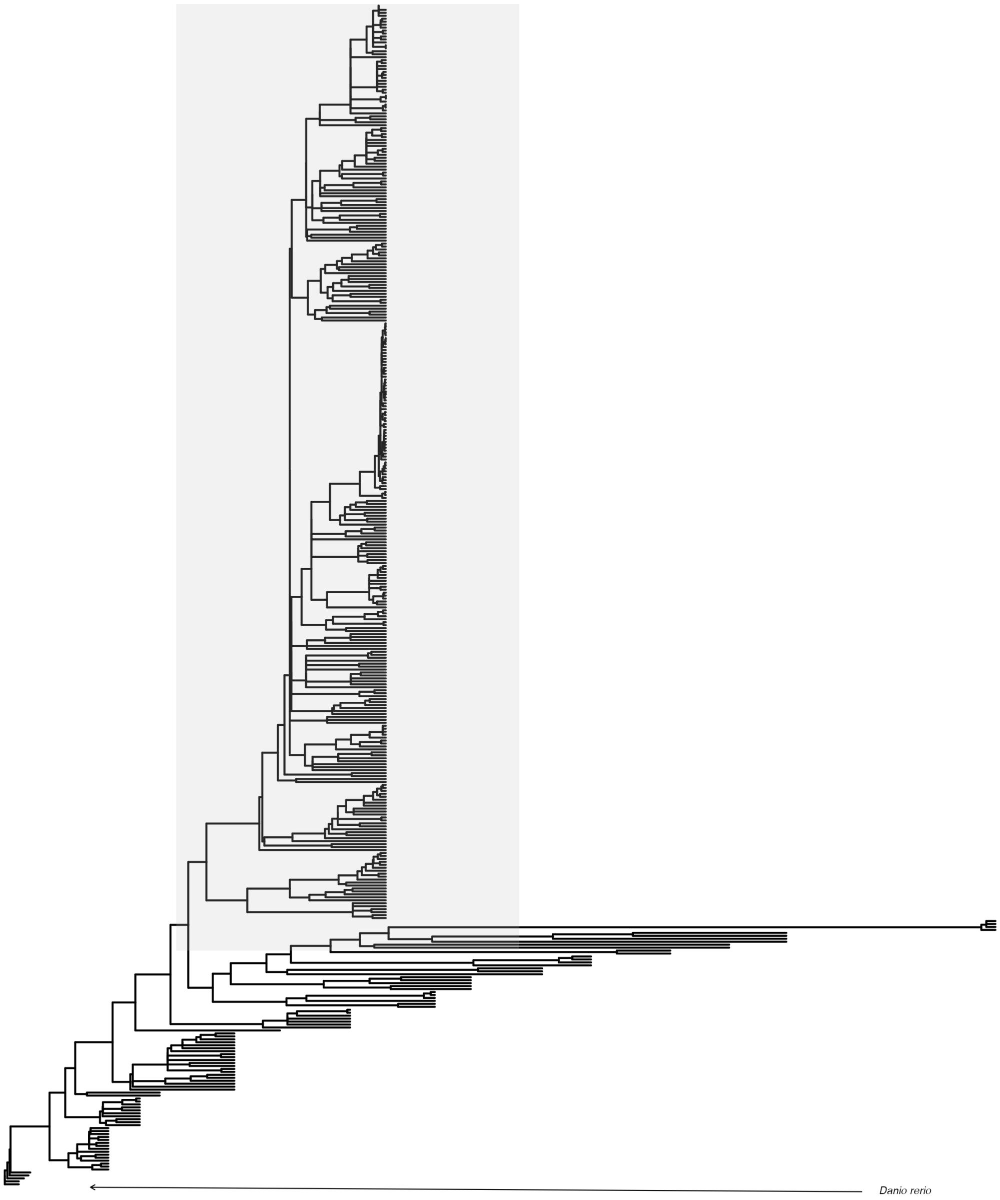

Figure S10. TimeTree for all fish species with lifespan and genomic information in our dataset. The tree shows estimated divergence of each species from the root species, zebrafish (*Danio rerio*) with no variability in values estimated for most species (highlighted in grey). Data is from http://www.timetree.org/.

MOLECULAR ECOLOGY

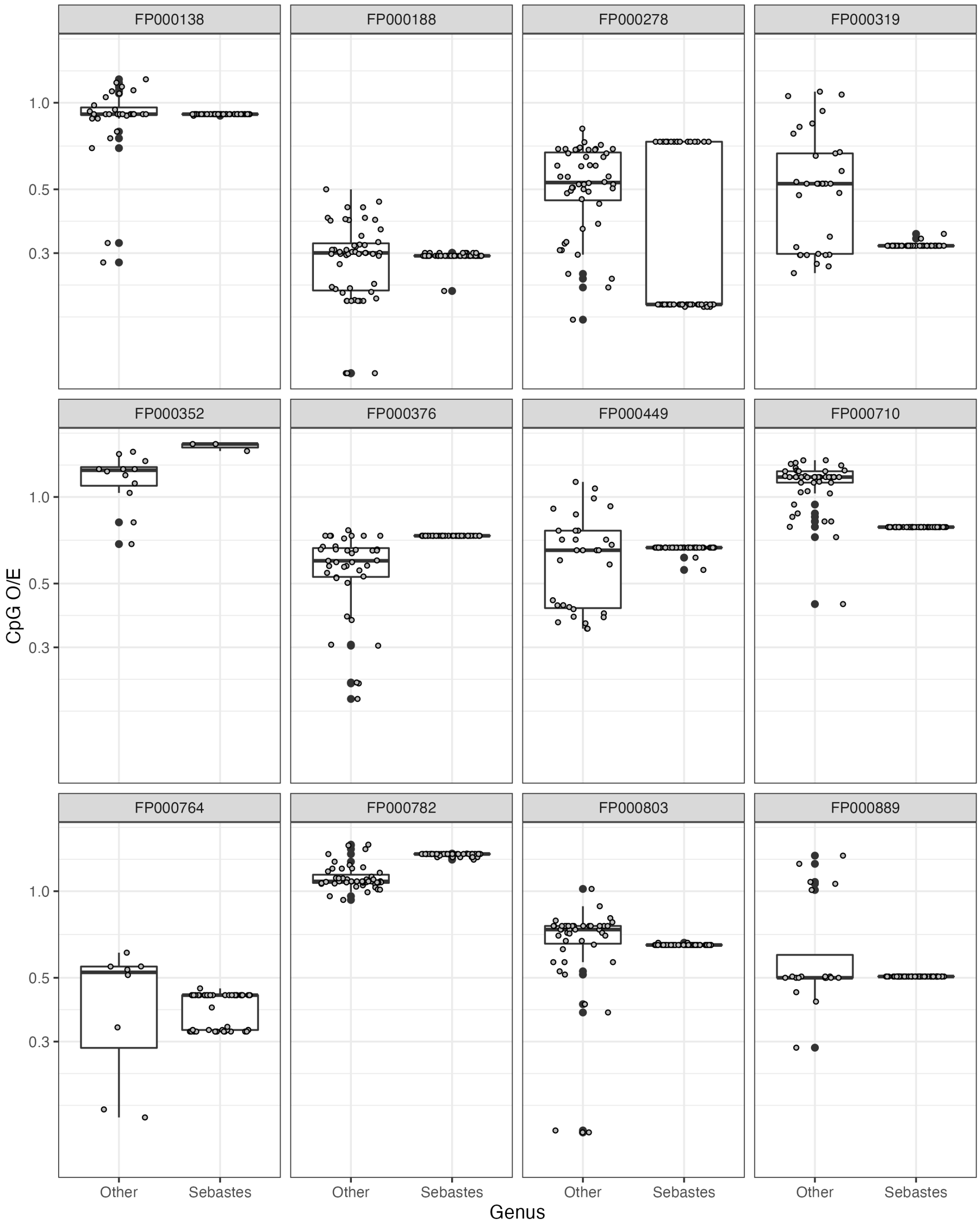

Figure S11. Lack of variation in *Sebastes* promoter CpG content. Box plots show CpG observed over expected (CpG O/E) values for the first 12 promoter regions (labelled FP######) present in the data set for all 57 *Sebastes* species and 57 fish species of other genera.

MOLECULAR ECOLOGY

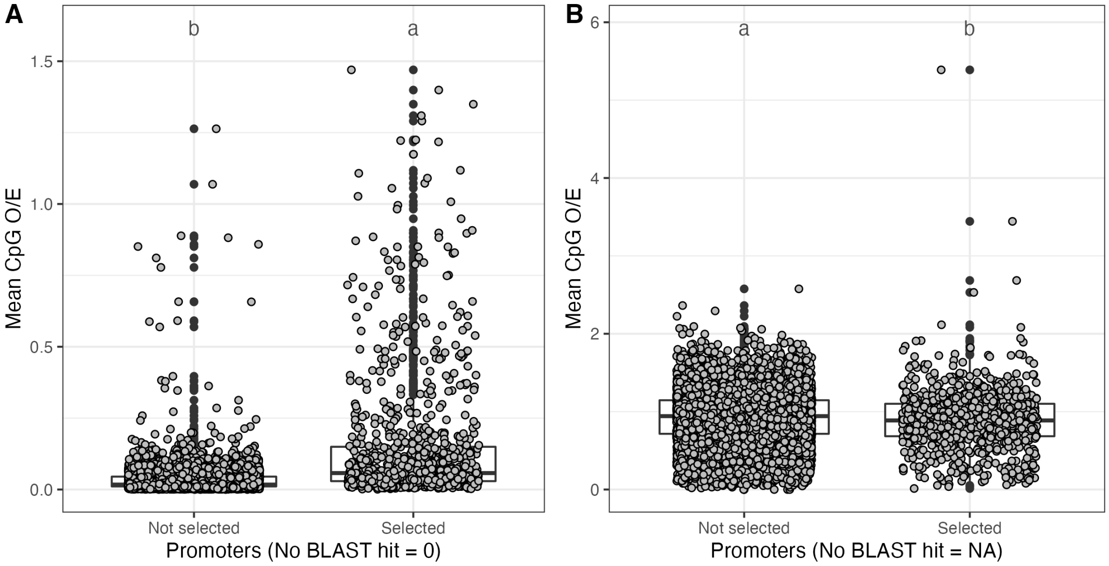

Figure S12. Mean CpG observed over expected ratio (CpG O/E) for each of the 10,230 zebrafish (*Danio rerio*) promoter regions used to build the fish lifespan predictor, based on whether they were selected during cross-validation of the final model ('Selected') or not ('Not selected'). **A.** For all values used in the model and; **B.** Where zero values derived from the absence of a BLAST hit are removed. Grey letters indicate results of a Welch's t-test.

MOLECULAR ECOLOGY

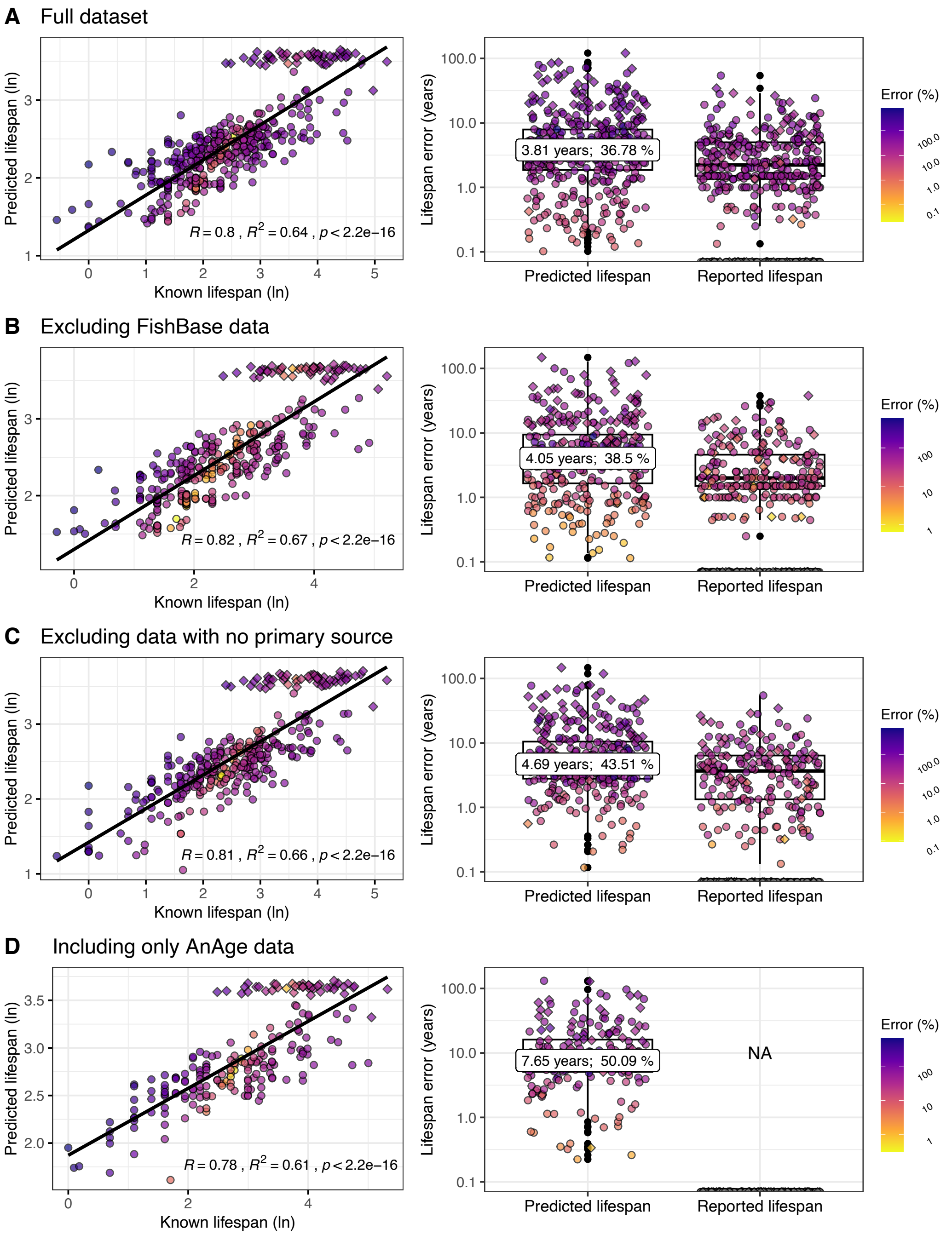

Figure S13. Comparison of model correlations between known and predicted lifespans (left) and the error in predicted and reported lifespans (right) for different data subsets. Error plots are labelled with median absolute and relative prediction error (in years and %, respectively). A. For the full dataset. B. Excluding all data derived from FishBase. C. Excluding data from any source for which no primary or original source information was available. D. Including only data from AnAge, a well curated database. Only one reported lifespan per species in D. meant that error could not be calculated ('NA'). Results indicate that the model produces the lowest median error when built using the full dataset (A). Information on error calculations is included in the corresponding figure in the main text. Diamond-shaped points indicate species from the *Sebastes* genus, circles indicate all others.

MOLECULAR ECOLOGY

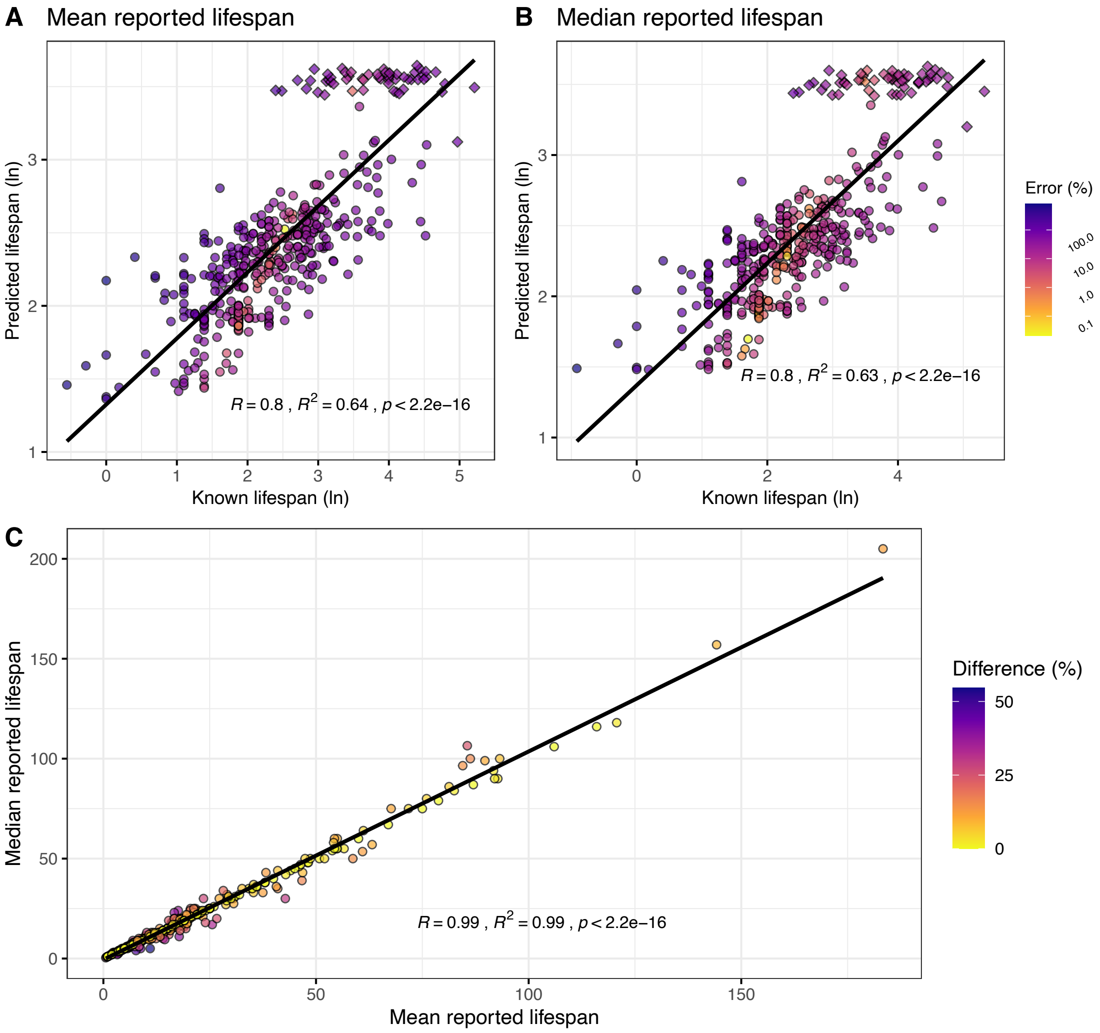

Figure S14. Comparison of model correlations between known and predicted lifespans when known lifespan is defined as A. the mean of all reported lifespans or B. the median of all reported lifespans for each species. C. Shows a direct comparison between the two known lifespan measures, showing little difference between the two. Information on error calculations is included in the corresponding figure in the main text. Diamond-shaped points indicate species from the *Sebastes* genus, circles indicate all others.

MOLECULAR ECOLOGY

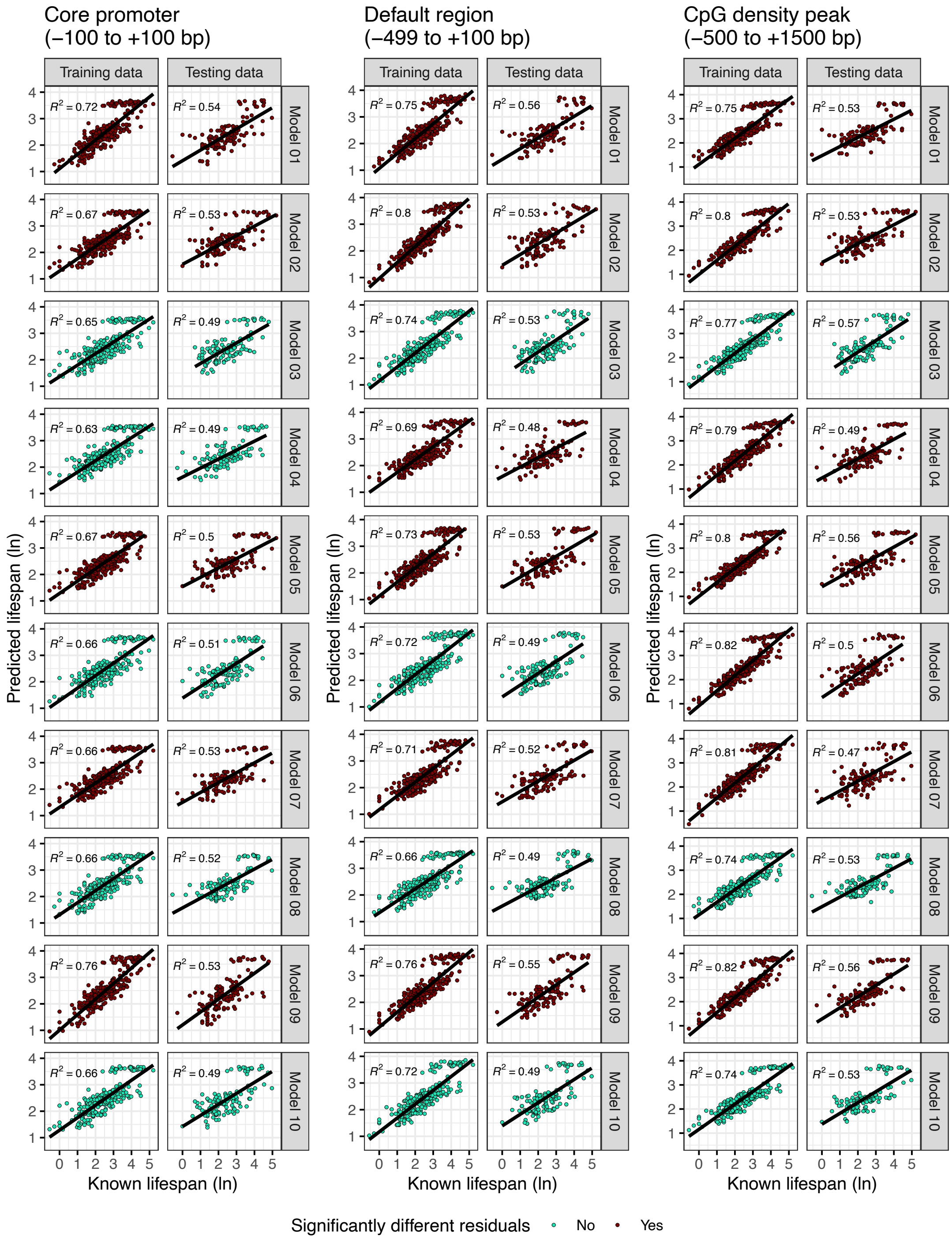

Figure S15. Comparison of 10-fold outer cross validation results for the fish lifespan model using different three promoter lengths (-100 to +100 bp, -499 to + 100 bp and -500 to +1500 bp, as titled). Plots show ccorrelations between known and predicted lifespans for the training and testing data sets (left and right columns), where the points are coloured according to the results of unpaired Students t-tests of the residuals (testing ~ training). The plot shows that the shortest ('core') promoter region produces the most consistent correlations between testing and training datasets.
